# A parallel randomised controlled trial of the Hypoglycaemia Awareness Restoration Programme for adults with type 1 diabetes and problematic hypoglycaemia despite optimised self-care (HARPdoc)

**DOI:** 10.1038/s41467-022-29488-x

**Published:** 2022-04-28

**Authors:** Stephanie A. Amiel, Laura Potts, Kimberley Goldsmith, Peter Jacob, Emma L. Smith, Linda Gonder-Frederick, Simon Heller, Elena Toschi, Augustin Brooks, Dulmini Kariyawasam, Pratik Choudhary, Marietta Stadler, Helen Rogers, Mike Kendall, Nick Sevdalis, Ioannis Bakolis, Nicole de Zoysa

**Affiliations:** 1 King’s College London, London, SE5 9RJ UK; 2 Department of Diabetes, King’s College Hospital NHS Foundation Trust, London, SE5 9RS UK; 3 Department of Biostatistics & Health Informatics, Institute of Psychiatry, Psychology and Neuroscience, King’s College London, London, UK; 4 University of Virginia School of Medicine, Charlottesville, VA USA; 5 University of Sheffield, Sheffield, S10 2TN UK; 6 Joslin Diabetes Center, Harvard Medical School, Boston, MA USA; 7 University Hospitals Dorset NHS Foundation Trust, Bournemouth, BH7 7DW UK; 8 Department of Diabetes and Endocrinology, Guy’s and St Thomas’ Hospital NHS Foundation Trust, London, SE1 9RT UK; 9 University of Leicester, Leicester, LE1 7RH UK; 10 HARPdoc Patient Group, Department of Diabetes, King’s College London, London, SE5 9RJ UK; 11 Centre for Implementation Science, King’s College London, London, SE5 8AF UK

**Keywords:** Endocrinology, Type 1 diabetes

## Abstract

Impaired awareness of hypoglycaemia (IAH) is a major risk for severe hypoglycaemia in insulin treatment of type 1 diabetes (T1D). To explore the hypothesis that unhelpful health beliefs create barriers to regaining awareness, we conducted a multi-centre, randomised, parallel, two-arm trial (ClinicalTrials.gov NCT02940873) in adults with T1D and treatment-resistant IAH and severe hypoglycaemia, with blinded analysis of 12-month recall of severe hypoglycaemia at 12 and/or 24 months the primary outcome. Secondary outcomes included cognitive and emotional measures. Adults with T1D, IAH and severe hypoglycaemia despite structured education in insulin adjustment, +/− diabetes technologies, were randomised to the “Hypoglycaemia Awareness Restoration Programme despite optimised self-care” (HARPdoc, n = 49), a psychoeducation programme uniquely focussing on changing cognitive barriers to avoiding hypoglycaemia, or the evidence-based “Blood Glucose Awareness Training” (BGAT, n = 50), both delivered over six weeks. Median [IQR] severe hypoglycaemia at baseline was 5[2–12] per patient/year, 1[0–5] at 12 months and 0[0–2] at 24 months, with no superiority for HARPdoc (HARPdoc vs BGAT incident rate ratios [95% CI] 1.25[0.51, 3.09], p = 0.62 and 1.26[0.48, 3.35], p = 0.64 respectively), nor for changes in hypoglycaemia awareness scores or fear. Compared to BGAT, HARPdoc significantly reduced endorsement of unhelpful cognitions (Estimated Mean Difference for Attitudes to Awareness scores at 24 months, −2.07 [−3.37,−0.560], p = 0.01) and reduced scores for diabetes distress (−6.70[−12.50,−0.89], p = 0.02); depression (−1.86[−3.30, −0.43], p = 0.01) and anxiety (−1.89[−3.32, −0.47], p = 0.01). Despite positive impact on cognitive barriers around hypoglycaemia avoidance and on diabetes-related and general emotional distress scores, HARPdoc was not more effective than BGAT at reducing severe hypoglycaemia.

## Abstract (web summary)

Impaired awareness of hypoglycaemia (IAH) is a risk for severe hypoglycaemia in insulin treatment of type 1 diabetes (T1D). Here the authors report that a group programme focussing on changing cognitive barriers to avoiding hypoglycaemia (HARPdoc) does not reduce severe hypoglycaemia more than a programme focussing on behaviours (BGAT) in a randomized control trial in adults with T1D and treatment-resistant IAH and severe hypoglycaemia.

## Introduction

Severe hypoglycaemia, defined as a blood glucose concentration so low that cognitive function is impaired and third-party assistance required^1^, remains a feared complication of insulin therapy for people with type 1 diabetes (T1D). There is an evidence-based pathway for minimising hypoglycaemia risk^2^. Structured education in flexible insulin therapy and developing technologies for glucose monitoring and insulin delivery have ameliorated the problem for some^3^ but not resolved it for all^4–9^. Access to technology remains patchy^10,11^ and successful engagement with technology where available is not universal^12–14^. Severe hypoglycaemia has many adverse biological, psychological and societal impacts, both acutely with each episode, and cumulatively over time^15^. More still needs to be done to reduce the risk of this feared complication of diabetes therapies.

The risk for severe hypoglycaemia is not evenly spread. In one study, 10% of people with T1D experienced nearly 70% of all episodes^16^. In a recent publication from a long-term follow-up of a large trial of intensified insulin therapy in T1D, while 54% of participants reported no severe hypoglycaemia (defined as coma or seizure) over 32 years, 8% reported more than five events^17^. Even among users of closed-loop insulin delivery at low risk of severe hypoglycaemia pre-system, 6% reported one or more episodes over six months of use.^18^ A key risk factor for severe hypoglycaemia is impaired awareness of hypoglycaemia (IAH)—loss of subjective awareness of a falling blood glucose in time to take action to avoid a severe episode^16,19^. IAH continues to be associated with significantly more severe hypoglycaemia in the era of continuous glucose monitoring.^20^

Cognitive factors are increasingly recognised as contributors to hypoglycemia risk and hypoglycaemia awareness status. Neuroimaging has shown differences in responses to hypoglycaemia in brain regions involved in emotional salience, reward, executive function and memory formation between people with and without hypoglycaemia awareness^21,22^. People with T1D and IAH express thoughts and beliefs around their hypoglycaemia that may be barriers against its avoidance^23^. Some at very high risk of severe hypoglycaemia express inappropriately low levels of concern^24^. People with IAH are less likely to use clinical advice about regimen adjustment than those in whom hypoglycaemia awareness remains intact^25^.

We had hypothesised that an intervention directly addressing unhelpful health beliefs about hypoglycaemia would be able to reduce severe hypoglycaemia and improve hypoglycaemia awareness in people with otherwise treatment-resistant problematic hypoglycaemia. We created and piloted an intervention that directly addressed the cognitive barriers described in the literature, which resulted in a marked reduction of severe hypoglycaemia at one year^26^. It was therefore timely to assess the intervention as an adjunct to existing therapies in a formal randomised controlled trial (RCT). We used patient and educator feedback to finalise our intervention, creating the “Hypoglycaemia Awareness Restoration Programme for adults with T1D and problematic hypoglycaemia despite optimised self-care” (HARPdoc)^27^, with specific focus on cognitive barriers to hypoglycaemia avoidance. Given the severity of the clinical problem being addressed, and the fact that the participants would all be under active clinical management, an inactive comparator intervention was not considered ethical. We therefore selected Blood Glucose Awareness Training (BGAT), a psycho-educational programme shown to improve awareness and reduce severe hypoglycaemia, but lacking the cognitive and psychotherapeutic elements of HARPdoc^28^, as a comparator intervention. BGAT has shown superiority in reducing severe hypoglycaemia over conventional care in an RCT format^29^ and is delivered over a time frame that could be adjusted to match the time frame for HARPdoc, facilitating randomisation of participants into either course, run concurrently. Despite its evidence base, the full English-language BGAT programme was not in clinical use at the time of the study and therefore not an existing treatment option.

Here we present an RCT of HARPdoc vs BGAT for problematic hypoglycaemia persisting despite otherwise optimised insulin self-management in adults with T1D. The primary endpoint was the rate of severe hypoglycaemia expressed per year at 12 and 24 months post randomisation, adjusted for baseline rates. Secondary endpoints included hypoglycaemia awareness scores; glycated haemoglobin (a measure of plasma glucose concentrations over the preceding two months) and the number of people achieving reductions in severe hypoglycaemia without elevation of this; endorsement of unhelpful cognitions around hypoglycaemia; fear of hypoglycaemia; diabetes distress and general mental health, reflected by anxiety and depression scores.

## Results

### Recruitment

Recruitment began in March 2017. The last participant was randomised in March 2019.

### Participants

**Fig. 1:**
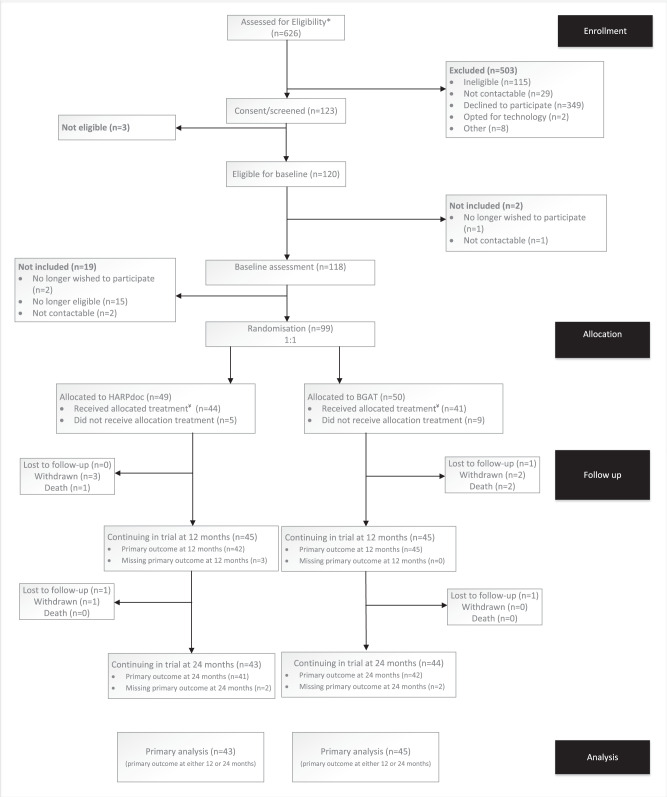
CONSORT diagram for the HARPdoc RCT, showing participant flow through the study. Participant numbers at each stage are shown in the figure. * Patients judged as potential participants for the trial, including some (*n* = 329) identified through remote review of electronic patient records by researchers followed by “cold call” and others identified at clinical team meetings. ¥ Attendance of at least the first 3 days of allocated treatment (HARPdoc or BGAT) plus at least one 1:1 session for HARPdoc was considered as “received allocated treatment”.

Of 626 people assessed for eligibility, 123 passed screening and consented to participate (Fig. 1). Of 349 people who declined participation, 329 had been identified in one centre by scanning electronic patient records, followed by “cold calling”. Decline rates were much lower in centres where potential participants had been identified in clinics and clinical team meetings. Mean(±SD) time from consent to the block randomisation was 7.8 ± 7.4 weeks, ranging from 3 days to 47 weeks. Baseline data were collected within one month of randomisation (1.9 ± 1.0 weeks, range 0–4.1) and courses then started within 3.1 ± 1.9 days. Eighty-three percent of participants were in UK centres (Table 1, Supplementary Table S1). Nine participants were withdrawn and three died, with no deaths associated with hypoglycaemia (Fig. 1, Supplementary data, Table S2).

**Table 1:** Baseline characteristics of participants in the HARPdoc RCT by intervention arm.

Demographic measure	HARPdoc	BGAT
*N*	49	50
*n* (%) in USA	8 (16.3)	9 (18.0)
Age (years)—mean ± sd	56.7 ± 12.2	51.9 ± 14.1
Female gender—*n* (%)	29 (59.2)	26 (52.0)
Ethnicity—*n* (%)		
White	46 (93.9)	49 (98.0)
African/Caribbean	0 (0.0)	1 (2.0)
Hispanic	1 (2.0)	0 (0.0)
Mixed	1 (2.0)	0 (0.0)
Other	1 (2.0)	0 (0.0)
Body Mass Index (BMI)—mean ± sd	[*N* = 48] 27.0 ± 5.5	[*N* = 50] 25.8 ± 4.4
Duration of type 1 diabetes (years)—mean ± sd	38.1 ± 16.0	33.6 ± 14.7
Glycated haemoglobin, HbA1c, %—mean ± sd	[*N* = 48] 7.4 ± 1.1	[*N* = 50] 7.4 ± 1.3
Glycated haemoglobin, HbA1c, mmol/mol—mean ± sd	[*N* = 48] 57.1 ± 12.2	[*N* = 50] 57.4 ± 14.1
Duration of experience of SH—*n* (%)		
<2 years	2 (4.1)	2 (4.0)
≥2 years to <5 years	14 (28.6)	8 (16.0)
≥5 years to <10 years	10 (20.4)	9 (18.0)
≥10 years	23 (46.9)	31 (62.0)
Baseline rate of SH—Median [IQR]	5.0 [2.0–11.0]	5.0 [2.0–12.0]
Diabetes complications and co-morbidities		
Pancreas transplant—*n* (%)	2 (4.1)	0 (0.0)
Islet transplant—*n* (%)	0 (0.0)	0 (0.0)
Renal transplant—*n* (%)	1 (2.0)	0 (0.0)
Symptomatic (peripheral) Neuropathy—*n* (%)	11 (22.4)	10 (20.0)
Retinopathy—*n* (%)	[*N* = 48] 34 (70.8)	[*N* = 50] 37 (74.0)
Number of comorbidities—mean ± sd	[*N* = 49] 7.2 ± 5.3	[*N* = 46]7.2 ± 5.0

*n* = number; *N* = number of participants with data.

### Allocation to intervention

Forty-nine participants were allocated to HARPdoc and 50 to BGAT (Fig. 1). These 99 participants form the population for the intention to treat (ITT) analysis. One randomised participant withdrew before the course started.

### Baseline characteristics

Table 1 provides the data by intervention group. The mean (±SD) age of the whole population was 54.3 ± 13.3 years, 56% female, 96% were white and BMI was 26.4 ± 4.9 kg/m^2^. Diabetes duration was long (35.8 ± 15.4 years), HbA1c reflected good control (7.4 ± 1.2%, 57.3 ± 13.1 mmol/mol). There was high co-morbidity (7.2 ± 5.1 significant additional medical conditions). By design, all participants had undertaken structured education in flexible insulin therapy, or, in a few cases, were judged by the local investigator as having had its equivalent, measured against a list of competencies (Table 2). Eighty-five percent (82.6% HARPdoc and 87.8% BGAT) had previously been offered some form of diabetes technology (pump, real-time continuous glucose monitoring (CGM) or both), with 55.6% (57.1% and 54.0%, respectively) using pump and/or CGM at baseline, randomisation successfully stratifying for this and for country (Table 2). The baseline rate of severe hypoglycaemia was, by design, high, albeit with a wide range (Table 3, columns 1 and 2 for baseline data by group). The number of severe hypoglycaemic episodes reported from the preceding year was 2891 (number with data, *N* = 99), with 599 reports of loss of consciousness or seizure (*N* = 91); 174 of glucagon or intravenous glucose treatment (*N* = 89); 105 ambulance call outs (*N* = 91); 38 Emergency Department visits (*N* = 91) and seven hospital admissions (*N* = 91). Gold and Clarke scores^30,31^ were high (mean ± SD 5.5 ± 1.2; 5.4 ± 1.1 respectively, shown by group in Table 4, data columns 1 and 2), confirming IAH. Over half the cohort (*n* = 54, 54.5%) had been experiencing problematic hypoglycaemia for over ten years (Table 1). Mean(±SD) score for diabetes distress, using the Problem Areas in Diabetes questionnaire (PAID), was not above the cut point for being high^32^ but had a wide range (31.9 ± 20.1). Scores in the Hospital Anxiety and Depression Score (HADS)^33^ were high^34^ (7.5 ± 4.6 and 5.9 ± 4.3, respectively), with 49.5 and 35.1% scoring 8 or more on the HADS-A and HADS-D respectively. Data by group are presented in data columns 1 and 2, Table 5. Fourteen HARPdoc (30.4%, *N* = 46) and 14 BGAT participants (30.4%, *N* = 46) were taking an antidepressant at baseline. In one HARPdoc and three BGAT participants, this had been started within the preceding two months. Seven participants (four HARPdoc, three BGAT) were in active treatment with a psychiatrist or psychologist.

**Table 2:** Experience at baseline of interventions in the therapeutic pathway for people with type 1 diabetes and problematic hypoglycaemia (education and technology) and stratification of randomisation in the HARPdoc RCT. N = numbers of participants with data.

	HARPdoc	BGAT
Current use of diabetes technology		
Insulin Pump—*n* (%)	[*N* = 47] 23 (48.9)	[*N* = 50] 22 (44.0)
Pump with automated suspend feature—*n* (%)	[*N* = 46] 8 (17.4)	[*N* = 50] 7 (14.0)
Continuous glucose monitoring (CGM)^a^— *n* (%)	[*N* = 47] 17 (36.2)	[*N* = 49] 17 (34.7)
Psychiatric or psychological therapies—*n* (%)	[N = 47] 4 (8.5)	[*N* = 50] 3 (6.0)
Retrospectively intermittently (Flash) glucose monitoring—*n* (%)	[*N* = 47] 3 (6.4)	[*N* = 50] 9 (18.0)
Currently using Bolus Advisor—*n* (%)	*[N* *=* *47] 27 (57.4)*	[*N* = 50] 16 (32.0)
Previous education in flexible insulin therapy		
Course attended—*n* (%)	[*N* = 46]	[*N* = 49]
DAFNE^b^	28 (57.1)	27 (54.0)
BERTIE^c^	6 (12.2)	10 (20.0)
DO IT^d^	4 (8.2)	4 (8.0)
Other structured education course	4 (8.2)	1 (2.0)
Other education	4 (8.2)	7 (14.0)
Unknown	3 (6.1)	1 (2.0)
Stratification factors by arm		
Technology user vs MDI and HBGM^e^—*n* (%)		
Technology	28 (57.1)	27 (54.0)
MDI and HBGM	21 (42.9)	23 (46.0)
Country of site—*n* (%)		
UK	41 (83.7)	41 (82.0)
USA	8 (16.3)	9 (18.0)

*MDI* multiple daily insulin injection regimen, *HBGM* home blood glucose monitoring (by fingerprick).

^a^Any real-time CGM device plus intermittently-monitored retrospective CGM (Flash) with additional on-line software providing real-time data and alarms.

^b^Dose Adjustment for Normal Eating^52^.

^c^Bournemouth Type 1 diabetes Education programme (https://www.uhd.nhs.uk/services/bdec/diabetes/structured-patient-education/bournemouth-type-1-diabetes-education-programme-bertie).

^d^Diabetes Outpatient Intensive Treatment Program (https://www.joslin.org/patient-care/education-programs-and-classes/do-it-program).

^e^Technology = insulin pump therapy and/or continuous glucose monitoring.

**Table 3:** Outcome descriptive statistics and effect estimates (Incidence rate ratios (IRR) from a three-level random intercept negative binomial model) with 95% Confidence intervals, for HARPdoc vs BGAT for primary and secondary endpoint data: hypoglycaemia episodes. Significance level of 0.025 (two sided) for primary outcome.

	HARPdoc baseline	BGAT baseline	HARPdoc 12 months	BGAT 12 months	IRR [95% CI]	*p*	HARPdoc 24 months	BGAT 24 months	IRR [95% CI]	*p*
Primary outcome measure										
Severe hypoglycaemia (SH) episodes in last 12 months M	*N* = 49	*N* = 50	*N* = 42	*N* = 45	1.25	0.62	*N* = 41	*N* = 42	1.26	0.64
Median(IQR)	5.0 (2.0–11.0)	5.0 (2.0–12.0)	1.0 (0.0–6.0)	1.0 (0.0–5.0)	[0.51, 3.09]		1.0 (0.0–2.0)	0.0 (0.0–2.0)	[0.48, 3.35]	
Primary data from open form, *n* (%)	5 (10.2)	4 (8.0)	10 (23.8)	16 (35.6)			4 (9.8)	10 (23.8)		
Secondary outcome measures										
Loss of consciousness or seizure	*N* = 45	*N* = 46	*N* = 32	*N* = 29	0.80	0.73	*N* = 37	*N* = 32	1.58	0.48
Median (IQR)	0.0 (0.0–2.0)	1.0 (0.0–3.0)	0.0 (0.0–1.0)	0.0 (0.0–2.0)	[0.23,2.84]		0.0 (0.0–1.0)	0.0 (0.0–0.0)	[0.44, 5.66]	
Glucagon or IV glucose	*N* = 44	N = 45	*N* = 32	N = 29	0.69	0.58	*N* = 37	*N* = 32	1.73	0.42
Median (IQR)	0.0 (0.0–2.5)	0.0 (0.0–1.0)	0.0 (0.0–1.0)	0.0 (0.0–1.0)	[0.18,2.59]		0.0 (0.0–1.0)	0.0 (0.0–0.0)	[0.46,6.57]	
Ambulance call out	*N* = 44	*N* = 46	*N* = 32	*N* = 29	0.46	0.19	*N* = 37	*N* = 32	2.67	0.15
Median (IQR)	0.0 (0.0–0.0)	0.0 (0.0–1.0)	0.0 (0.0–0.0)	0.0 (0.0–0.0)	[0.14,1.46]		0.0 (0.0–0.0)	0.0 (0.0–0.0)	[0.71,10.00]	
A and E attendance	*N* = 45	*N* = 46	*N* = 32	*N* = 29	2.21	0.48	*N* = 37	*N* = 32	1.17	0.90
Median (IQR)	0.0 (0.0–0.0)	0.0 (0.0–0.0)	0.0 (0.0–0.0)	0.0 (0.0–0.0)	[0.24,20.26]		0.0 (0.0–0.0)	0.0 (0.0–0.0)	[0.09,15.10]	
Hospital admission	*N* = 45	*N* = 46	*N* = 32	*N* = 29	Unable to analyse due to low number of events		*N* = 37	*N* = 32	Unable to analyse due to low number of events	
Median (IQR)	0.0 (0.0–0.0)	0.0 (0.0–0.0)	0.0 (0.0–0.0)	0.0 (0.0–0.0)			0.0 (0.0–0.0)	0.0 (0.0–0.0)		
Moderate hypoglycaemia, episodes in last 4 weeks, total	*N* = 47	*N* = 47	*N* = 41	*N* = 41	1.03	0.93	*N* = 34	*N* = 36	1.01	0.97
Median (IQR)	5.0 (2.0–8.0)	10.0 (3.0–19.0)	3.0 (2.0–10.0)	5.0 (2.0–10.0)	[0.52,2.04]		3.0 (1.0–9.0)	4.0 (1.0–10.5)	[0.49,2.30]	

*BGAT* Blood Glucose Awareness Training, *HARPdoc* Hypoglycaemia Awareness Restoration Programme for people with T1D and problematic hypoglycaemia despite optimised control, *N* number of participants with data.

**Table 4:** Outcome descriptive statistics and effect estimates (mean difference/odds ratio [OR] from a three-level random intercept linear/logistic regression model) with 95% Confidence intervals, for HARPdoc vs BGAT for secondary endpoint data: other glucose-related outcomes. p values for significance <0.05 or <0.025 with correction for multiple comparisons (Bonferoni), two sided.

	HARPdoc Baseline	BGAT Baseline	HARPdoc 12 months	BGAT 12 months	Estimated mean difference [95% CI]	*p*	HARPdoc 24 months	BGAT 24 months	Estimated mean difference [95% CI]	*p*
Gold scores	*N* = 48	*N* = 49	*N* = 41	*N* = 45	−0.48	0.11	*N* = 39	*N* = 40	−0.27	0.37
Mean ± sd	5.5 ± 1.1	5.1 ± 1.2	4.2 ± 1.5	4.3 ± 1.8	[−1.07,0.11]		3.8 ± 1.6	3.9 ± 1.9	[−0.87,0.33]	
Clarke scores	*N* = 48	*N* = 50	*N* = 41	N = 45	−0.10	0.72	*N* = 39	*N* = 41	0.28	0.33
Mean ± sd	5.6 ± 0.8	5.2 ± 1.2	4.5 ± 1.4	4.4 ± 1.6	[−0.66,0.46]		4.3 ± 1.6	3.8 ± 1.7	[−0.29,0.85]	
Glycated Haemoglobin %	*N* = 48	*N* = 50	*N* = 41	N = 43			*N* = 35	*N* = 41		
Mean ± sd	7.4 ± 1.1	7.4 ± 1.3	7.3 ± 1.1	7.5 ± 1.0			7.4 ± 1.0	7.5 ± 1.3		
Glycated haemoglobin mmol/mol	*N* = 48	*N* = 50	*N* = 41	*N* = 43	−0.05	0.69	*N* = 35	*N* = 41	−0.10	0.47
Mean ± sd	57.1 ± 12.2	57.4 ± 14.1	56.7 ± 11.8	58.0 ± 11.2	[−0.30,0.20]		56.8 ± 10.9	57.9 ± 14.3	[−0.38,0.17]	
					OR [95% CI]				OR [95% CI]	
Those with HbA1c rise ≤0.3%,			*N* = 41	*N* = 43	1.26	0.78	*N* = 35	*N* = 34	4.94	0.11
*n* (%)			28 (68.3)	28 (65.1)	[0.25,6.29]		28 (80.0)	22 (64.7)	[0.70,34.72]	

*BGAT* Blood Glucose Awareness Training, *HARPdoc* Hypoglycaemia Awareness Restoration Programme for people with T1D and problematic hypoglycaemia despite optimised control, *n* number; *N* number of participants with data.

### Progress through trial

The mean(±SD) duration of follow-up in the trial was 737.7 ± 31.7 days (range 562–827). Time in trial for the 12-month follow-up was 375.2 ± 31.2 days (range 310–546) and 737.7 ± 31.7 (range 562–827) for 24 months. Twenty-three participants met one or more protocol violations and were removed from the per protocol (PP) analysis (Fig. 1, supplementary data, table S2, lower panel).

### Primary outcome (top, Table 3, Fig. 2 with Table 5)

**Fig. 2:**
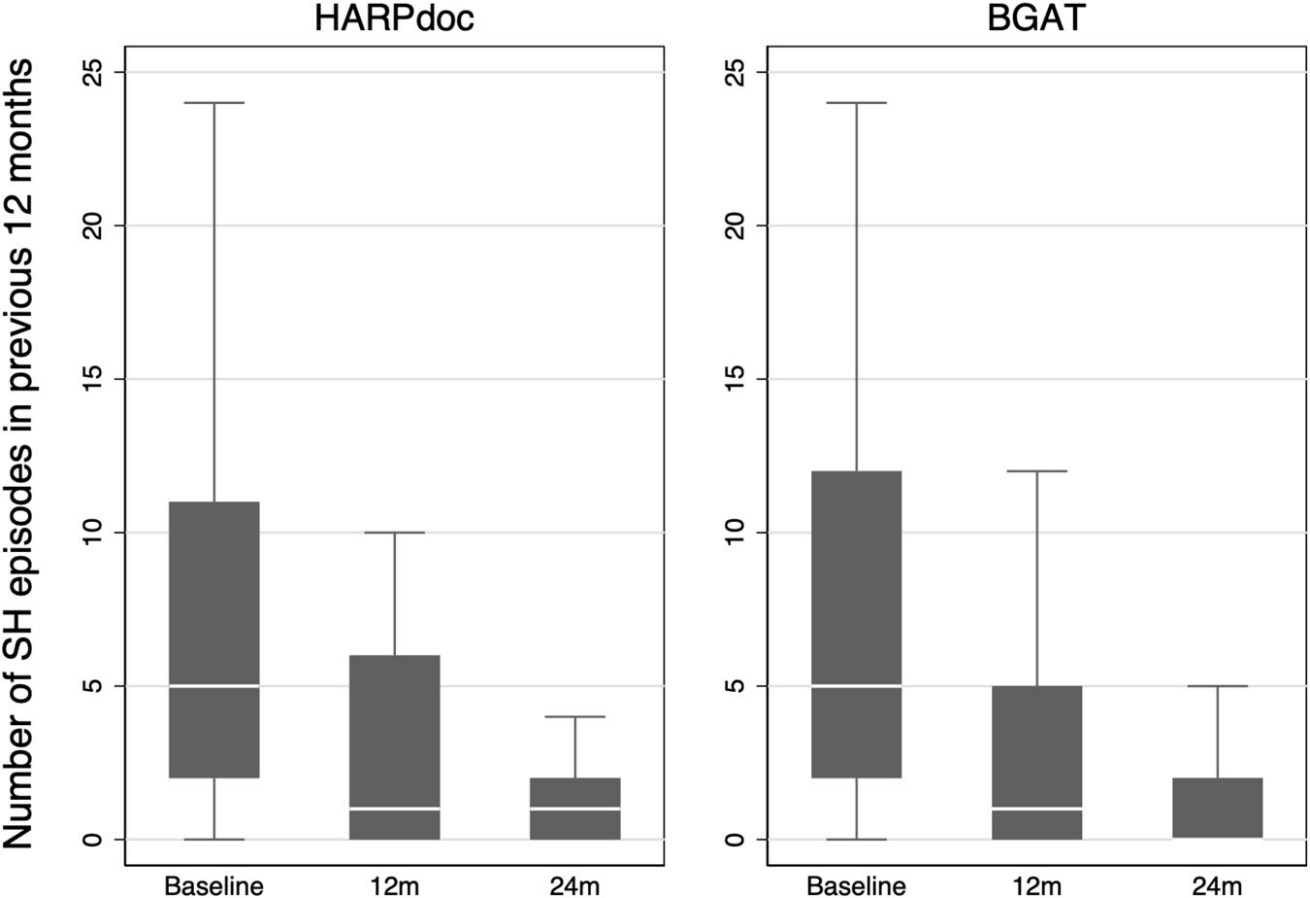
A box plot to present the summary statistics of the number of SH events in the previous 12 months (primary endpoint) by treatment arm and time point. The grey box represents the 25–75% interquartile range, the median is represented by the white line in each box and the whiskers cover the minimum to the maximum values, to the limit of 1.5× the IQR. Individual values for rates outside this limit have been excluded for clarity and are presented in Table 5. A three-level random intercept negative binomial model has been used, using a missing at random assumption (MAR) and a significance level of 2.5% (two sided). The N for each group is baseline: 49 and 50, 12 months 42 and 45 and 24 months 41 and 42 HARPdoc and BGAT respectively. Source data are available on request to SAA or IB, as described in the text.

Data on the primary endpoint were available for 87 participants at 12 months and 83 at 24 months. There were 752 severe hypoglycaemia events recalled over the previous 12 months at 12 months (501 HARPdoc, 200 from one participant, *n* = 42; 251 BGAT, *n* = 45) and 349 at 24 months (76 HARPdoc, n = 41 and 273 BGAT, *n* = 42). In the ITT analysis, median [interquartile range] severe hypoglycaemia episodes were 5.0 [2–12] at baseline; 1.0 [0.0–5.0] at 12 months and 0.0 [0.0–2.0] at 24 months, all per patient per year. There were no significant differences, adjusted for baseline, between the two groups, with incidence rate ratio (IRR) [95% CI], HARPdoc vs BGAT of 1.25 [0.51, 3.09], p = 0.62 and 1.26 [0.48, 3.35], *p* = 0.64 at 12 and 24 months respectively (Fig. 2 and Table 5). The percent of people with >1 severe hypoglycaemia in 12 months at baseline, 12 and 24 months respectively fell from 83.7% to 12.0 and 1.85% in HARPdoc, and from 76. 5 to 6 and 6.5% in BGAT. Seventeen percent of primary outcome data were missing, with only female gender of key baseline characteristics associated with missingness (*p* < 0.01). A post randomisation measure of course completion was also found to predict missingness (*p* = 0.01). Accounting for missing data biases using multiple imputation by chained equations (MICE) did not alter the outcome (IRRs 1.33[0.53,3.31], *p* = 0.55 and 1.16[0.43–3.10], *p* = 0.76), nor did including use of real-time CGM (any rtCGM plus Libre 1 with Miao Miao software) as an interaction term of the intervention (HARPdoc vs BGAT) with rtCGM across the two time points present any interaction effect (*p* = 0.115). The PP analysis also showed no between-group differences (IRR 1.13[0.35,3.71], *p* = 0.81, *n* = 67 and 1.04[0.30,3.61], *p* = 0.94, *n* = 65). A pre-specified subgroup analysis did not provide evidence that the effect of HARPdoc vs BGAT was moderated in participants expressing low worry (<0.92) or low behaviour (<1.85) scores in the HFS-II survey (supplementary data Table S3). The interaction in the model showed no evidence of a difference for both HFS-II behaviour subgroups (*p* = 0.214) and worry subgroups (*p* = 0.421).

**Table 5:** Numbers of severe hypoglycaemia episodes recalled over previous 12 months by individual participants that exceed 1.5× the interquartile range for the group at each main time point in the trial.

HARPdoc			BGAT		
Episodes of severe hypoglycaemias recalled over previous 12 months not presented in Fig. 2			Episodes of severe hypoglycaemias recalled over previous 12 months not presented in Fig. 2		
Baseline	12 m	24 m	Baseline	12 m	24 m
552	200	12	250	100	180
500	104	10	208	32	40
300	80	8	208	29	12
52	24	6	100		9
52	17	6	51		8
40			34		
29			30		
			30		

These numbers are excluded from the representation of the data in Fig. 2 for clarity.

### Adjudication of severe hypoglycaemic events

Four hundred and twenty-seven reports of severe hypoglycaemia submitted at the time, or within the calendar month after, an event were sent for adjudication. Of these, 89.5% were considered definite or probable severe hypoglycaemia by two independent adjudicators.

### Secondary endpoints

Outcomes for secondary endpoints related to glycaemia are also shown in Table 3. In addition to total events over the preceding 12 months, participants reported specific adverse outcomes of severe hypoglycaemia at 12 and 24 months on anonymised forms. During the 24-month follow-up, 218 episodes were reported with loss of consciousness or seizure (131 HARPdoc, 87 BGAT); 156 events treated with intravenous glucose or intramuscular glucagon (72 HARPdoc, 84 BGAT); 73 ambulance call outs (52 HARPdoc, 21 BGAT); 19 attendances at Accident and Emergency/Emergency Departments (14 HARPdoc, 5 BGAT) and 2 hospital admissions including one night or more (0 HARPdoc, 2 BGAT). Median rates per participant per year for these events were very low, mostly 0. There were no significant differences in these outcomes between the interventions, IRRs ranging from 0.46 to 2.67, all non-significant (Table 3). Moderate hypoglycaemia event rates were not different at 12 or 24 months between interventions. There were also no significant between-group differences in Gold or Clarke hypoglycaemia awareness scores at either 12 or 24 months, estimated mean differences −0.48 to 0.28 (Table 4).

Neither HbA1c measured biochemically nor the proportion of people in whom HbA1c did not rise by more than 0.3% from its starting value at 12 and 24 months differed between groups (Table 4).

**Fig. 3:**
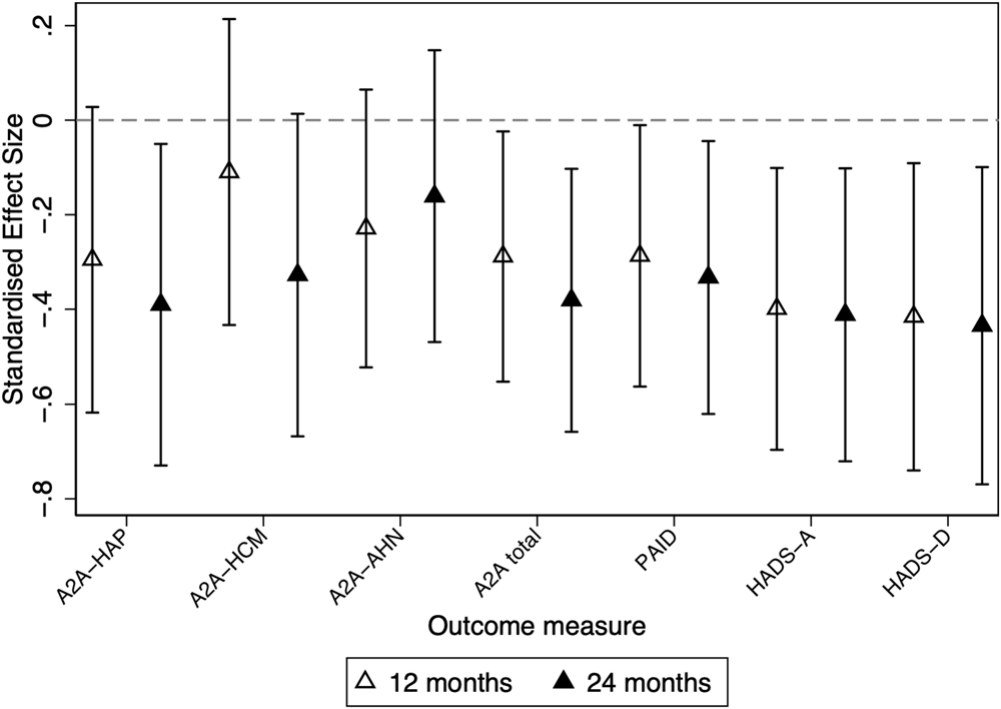
Standardised effect sizes of secondary endpoints A2A, PAID and HADS. Values below zero indicate better outcomes in HARPdoc. The data are presented as estimated mean differences and 95% confidence intervals, with open triangles representing means of 12 month data; closed triangles representing means of 24-month data and bars representing 95% confidence intervals. Analysis is by a three-level random intercept linear regression regression model. A significance level of 5% (two sided) has been used. The standardised effect sizes for other secondary endpoints were not significant and are shown, together with numerical data for the depicted parameters, and the numbers providing data at each time point in each intervention, in Tables 4 and 6. Source data are available on request to S.A.A. or I.B., as described in the text.

Data on cognitive, emotional and mental health outcomes are presented in Tables 6 and 7 and Fig. 3. There was differential impact of the two interventions on some non-glucose outcomes. Reflecting the degree to which participants endorsed cognitions that might form barriers to avoidance of future hypoglycaemia, the Attitudes to Awareness total scores were lower in HARPdoc compared to BGAT at both 12 and 24 months (mean ± SD 7.6 ± 4.6 vs 8.7 ± 4.4, *p* = 0.03 and 7.1 ± 4.2 vs 8.9 ± 3.4, *p* = 0.01 HARP vs BGAT at 12 and 24 months, respectively). Likewise, the A2A factor “Hyperglycaemia Avoidance Prioritised” score was different between interventions by 24 months (4.6 ± 2.9 vs 5.1 ± 2.3, *p* = 0.02). Differences in the total A2A score at 12 months between HARPdoc and BGAT did not remain significant after correction for multiple comparisons. Differences between the groups on “Hypoglycaemia concern minimised” and “Asymptomatic hypoglycaemia normalised” scores did not reach significance.

**Table 6:** Outcome descriptive statistics and effect estimates (mean difference from a three-level random intercept linear regression model) with 95% Confidence intervals for HARPdoc vs BGAT for secondary endpoint data: cognitive outcomes (the Attitudes to Awareness questionnaire, A2A) and worry and behaviours (Hypoglycaemia Fear Score II (HFS-II) and Hyperglycaemia Avoidance Survey (HAS)).

	HARPdoc baseline	BGAT baseline	HARPdoc 12 month	BGAT 12 month	Estimated mean difference [95% CI]	*p*	HARPdoc 24 month	BGAT 24 month	Estimated mean difference [95% CI]	*p*
A2A Factor: Hyperglycaemia Avoidance Prioritised	*N* = 47	*N* = 50	*N* = 42	*N* = 44	0.08	0.07	*N* = 37	*N* = 40	−1.06	0.02
Mean ± SD	6.1 ± 2.9	5.6 ± 2.5	4.6 ± 2.9	5.1 ± 2.3	[−1.67,0.08]		4.0 ± 2.5	4.9 ± 2.1	[−1.97,−0.14]	
A2A Factor: Hypoglycaemia Concern Minimised	*N* = 47	*N* = 50	*N* = 42	*N* = 44	−0.21	0.51	*N* = 37	N = 40	−0.62	0.06
Mean ± SD	2.5 ± 2.0	2.4 ± 1.7	2.2 ± 1.5	2.4 ± 1.7	[−0.82,0.40]		1.9 ± 1.5	2.7 ± 2.0	[−1.26,0.03]	
A2A Factor: Asymptomatic Hypoglycaemia Normalised	*N* = 47	N = 50	*N* = 42	N = 44	−0.50	0.13	*N* = 37	N = 40	−0.35	0.31
Mean ± SD	2.1 ± 2.5	1.4 ± 1.8	0.9 ± 1.5	1.2 ± 1.9	[−1.15,0.14]		1.1 ± 1.6	1.3 ± 1.7	[−1.03,0.33]	
A2A total score	*N* = 47	*N* = 50	*N* = 42	*N* = 44	−1.56	0.03	*N* = 37	*N* = 40	−2.07	0.01
Mean ± SD	10.8 ± 6.2	9.3 ± 4.6	7.6 ± 4.6	8.7 ± 4.4	[−3.00.−0.13]		7.1 ± 4.2	8.9 ± 3.4	[−3.57,−0.56]	
HFS-II Behaviour subscale	*N* = 47	*N* = 50	*N* = 41	*N* = 44	−0.18	0.10	*N* = 36	*N* = 40	−0.05	0.69
Mean ± SD	1.5 ± 0.8	1.4 ± 0.8	1.2 ± 0.8	1.4 ± 0.8	[−0.40,0.03]		1.2 ± 0.8	1.4 ± 0.8	[−0.27, 0.18]	
HFS-II Worry subscale	*N* = 47	*N* = 50	*N* = 42	*N* = 44	0.003	0.98	*N* = 361	*N* = 40	0.08	0.54
Mean ± SD	1.7 ± 0.9	1.7 ± 1.0	1.5 ± 0.7	1.5 ± 1.0	[−0.23,0.24]		.2 ± 0.8	1.3 ± 1.0	[−0.17,0.33]	
HFS-II Total score	*N* = 47	*N* = 50	*N* = 41	*N* = 44	−0.08	0.45	N = 36	N = 40	0.03	0.77
Mean ± SD	1.6 ± 0.8	1.6 ± 0.8	1.4 ± 0.7	1.5 ± 0.8	[−0.27,0.12]		1.2 ± 0.7	1.4 ± 0.9	[−0.17,0.23]	
HAS Behaviour Subscale	*N* = 46	*N* = 50	*N* = 42	*N* = 44	−0.16	0.09	N = 37	N = 38	−0.07	0.46
Mean + SD	1.8 ± 0.6	1.8 ± 0.6	1.5 ± 0.6	1.7 ± 0.6	[−0.35,0.03]		1.5 ± 0.5	1.5 ± 0.5	[−0.27,−0.12]	
HAS Worry Subscale	*N* = 47	*N* = 50	*N* = 42	*N* = 44	−0.19	0.09	N = 37	N = 40	−0.23	0.05
Mean ± SD	2.0 ± 0.7	1.9 ± 0.6	1.7 ± 0.7	1.9 ± 0.7	[−0.41,0.03]		1.7 ± 0.7	1.9 ± 0.6	[−0.46, −0.003]	
HAS total score	*N* = 46	*N* = 50	*N* = 42	*N* = 44	−0.18	0.05	N = 37	N = 39	−0.16	0.11
Mean ± SD	1.9 ± 0.5	1.9 ± 0.5	1.6 ± 0.6	1.8 ± 0.5	[−0.37,−0.0009]		1.6 ± 0.5	1.7 ± 0.5	[−0.35,0.04]	

*P* values for significance, <0.05, <0.025 with correction for multiple comparisons, two sided. *N* = number of participants with data.

**Table 7:** Outcome descriptive statistics and effect estimates (mean difference from a three-level random intercept linear regression model) with 95% Confidence intervals for HARPdoc vs BGAT for secondary endpoint data: mental health questionnaires (Diabetes Distress (PAID); Hospital Anxiety and Depression scores (HADS-A and HADS-D)).

	HARPdoc baseline	BGAT baseline	HARPdoc 12 month	BGAT 12 month	Estimated mean difference [95% CI]	p	HARPdoc 24 month	BGAT 24 month	Estimated mean difference [95% CI]	*p*
PAID, Total score	*N* = 46	*N* = 50	N = 42	*N* = 44	−5.77	0.04	*N* = 37	*N* = 40	−6.70	0.02
Mean ± SD	33.2 ± 21.3	30.6 ± 19.1	26.9 ± 19.3	31.9 ± 19.6	[−11.34,−0.21]		21.2 ± 18.5	29.1 ± 20.0	[−12.50,−0.89]	
Anxiety (HADS-A)	*N* = 47	*N* = 50	N = 42	*N* = 44	−1.84	0.01	*N* = 37	*N* = 40	−1.89	0.01
Mean ± SD	7.3 ± 4.6	7.8 ± 4.6	5.9 ± 4.5	8.4 ± 5.3	[−3.21,−0.47]		5.1 ± 4.1	8.2 ± 5.2	[−3.32,−0.47]	
Anxiety (HADS-A)										
No (%) scoring ≥8	20 (42.6)	28 (56.0)	14 (33.3)	25 (56.8)			11 (29.7)	20 (50.0)		
Depression (HADS-D)	*N* = 47	*N* = 50	N = 42	*N* = 44	−1.78	0.01	*N* = 37	*N* = 40	−1.86	0.01
Mean ± SD	5.5 ± 4.6	6.3 ± 3.9	4.4 ± 4.3	6.8 ± 4.6	[−3.17,−0.39]		4.1 ± 3.6	7.3 ± 5.5	[−3.30,−0.43]	
Depression (HADS-D)										
No (%) scoring ≥8	15 (31.9)	19 (38.0)	11 (26.2)	19 (43.2)			8 (21.6)	17 (42.5)		

*P* values for significance, <0.05, <0.025 with correction for multiple comparisons, two sided. *N* = number of participants with data.

There were differences between the two interventions, with HARPdoc showing superiority, in the PAID scores of diabetes distress (26.9 ± 19.3 vs 31.9 ± 19.6, *p* = 0.04 and 21.2 ± 18.5 vs 29.1 ± 20.0, *p* = 0.02, HARPdoc vs BGAT, 12 and 24 months, respectively), and in scores for anxiety and depression, measured by the HADS-A (5.9 ± 4.5 vs 8.4 ± 5.3, *p* = 0.01 and 5.1 ± 4.1 vs 8.2 ± 5.2, *p* = 0.01) and HADS-D (4.4 ± 4.3 vs 6.8 ± 4.6, *p* = 0.01 and 4.1 ± 3.6 vs 7.3 ± 5.5, *p* = 0.01) questionnaires. All differences in the PAID, HADS-A and HADS-D scores remained significant after correction for multiple comparisons, except for the PAID score at 12 months. These differences were significant both for those with complete data and when accounting for missing data using MICE (Supplementary Table S4a, b). Total, worry and behaviour scores on the hyperglycaemia avoidance or hypoglycaemia fear (HSF-II) questionnaires did not differ between groups (Table 6).

Although not a stated secondary outcome, the analysis plan included summarising new uptake of technology, as being likely to impact severe hypoglycaemia. During the trial, uptake - either offered by local care teams unrelated to the trial or in discussion with the investigators - was limited, with fewer than 30% of participants starting to use a technology (CGM, pump, automated suspension of insulin delivery and/or bolus dose advisor functions) by the end of follow-up (Supplementary Table S5). Re-running our analysis models excluding the five HARPdoc and nine BGAT participants who started any form of real-time CGM during their follow-up did not affect our outcomes, with all *p* values for interaction >0.1.

New psychological therapies were started by 12 months in one of 42 HARPdoc and six of 44 BGAT participants (2.4% and 13.6%, respectively), with a further two of 37 HARPdoc and three of 40 BGAT new users in each group (5.4% and 7.5%, respectively) by 24 months. New prescriptions of psychotropic medications were started by two each of 46 HARPdoc and 43 BGAT participants (4.3 and 4.7% respectively) by 12 months, with a further two participants starting them in each group (4.4%, *N* = 45 and 5%, *N* = 40, respectively) by 24 months.

Adverse event (AE) data are provided in Supplementary Table S6. There were 45 AEs in 27 people and 21 serious adverse events (SAEs) in 14 people in total. Three SAEs resulted in death, two cardiovascular and one subdural haemorrhage. There were no AEs or SAEs that were possibly or definitely related to study interventions. One participant reported Covid-19 infection, not classified as serious, with full recovery.

We examined the data for an impact of the Covid-19 pandemic. The 12-month data collection for the last-entered participants was completed in London on 12^th^ March 2020, before any major societal impact of the pandemic. Data collection for outstanding visits continued during Covid-19 restrictions, with participants accessing questionnaires online, collecting blood at home and completing visits by telephone or video-conference, or face to face when permitted, according to participant preference. Outcome data collected before Covid restrictions (*n* = 30) and after (*n* = 69) are given in supplementary data table S7. There was no evidence of an impact of having provided data during the Covid pandemic on the primary endpoint (*p* = 0.997) and no differences in 24-month outcome measures between secondary outcome data collected before and during Covid-19 restrictions, apart from a slight imbalance in the A2A Hyperglycaemia Avoidance Prioritised subscale and the A2A total score (supplementary data, table S8).

## Discussion

The HARPdoc RCT successfully recruited 99 adults with T1D and treatment-resistant problematic hypoglycaemia and randomised them to either the HARPdoc intervention, with its focus on cognitive restructuring, or the pre-existing, updated, BGAT intervention, with its focus on behavioural training. While, as might have been expected, severe hypoglycaemia rates were lower in both groups at 12 and 24 compared to baseline, there was no significant difference between the groups. Although the trial did not show superiority for HARPdoc for this endpoint, analysis of secondary outcome data indicate that HARPdoc was superior to BGAT in altering unhelpful health beliefs around hypoglycaemia considered to be barriers to avoidance of future hypoglycaemia. HARPdoc was also superior in reducing scores measuring diabetes distress, anxiety and depression.

Severe hypoglycaemia and IAH (a key secondary endpoint) are not trivial side-effects of insulin therapy. The former may be associated with confusion, abnormal behaviour, injury, coma, seizure and occasionally death and both conditions have negative impact on quality of life for people with diabetes and their families, as well as economic impact^15^. The participants recruited into our study were intended to be people at highest risk, by virtue of their current experience of severe episodes^35,36^ and their IAH^15,20,35,36^. In line with our intention, the demographic characteristics of our cohort reflects both known non-modifiable risks for severe hypoglycaemia (long diabetes duration and high co-morbidity^15,35^) and modifiable risks (recurrent severe hypoglycaemia^35^ and IAH^19,20,36^). The participants were also people whose hypoglycaemia problems had persisted despite appropriate conventional interventions^2,3^. All our participants had undertaken education in flexible insulin therapy, the intervention with greatest impact on severe hypoglycaemia rates^3,37^ and most had been offered diabetes technologies, with over half using them at randomisation. Closed-loop insulin delivery systems were not available to participants at recruitment but those of its component technologies with proven benefit on severe hypoglycaemia (continuous glucose monitoring and automated suspension of insulin delivery^9,38^) were available. Our data suggest that this cohort may not have been able to use them successfully prior to the trial^39^. The cohort thus represents a highly vulnerable, hitherto treatment-resistant, population, unable to gain benefit from existing strategies for hypoglycaemia risk reduction, who will be very familiar to diabetes health care professionals. While it is possible that further improvements in technology, such as greater accuracy or less obtrusiveness, may be helpful to this group, it remains likely that human factors will remain important in maximising the number of people who can gain full benefit. For most of the participants in the present trial, the reduction in severe hypoglycaemia occurred without change in technology usage. Outcomes were not different for users of rtCGM and removing the data of people who started rtCGM during follow-up (slightly more in BGAT) did not alter outcomes. Nevertheless, better engagement with technology would be a positive outcome of the courses—the interventions tested here are adjuncts to current hypoglycaemia management pathways, not replacements for their other elements.

The unique feature of the HARPdoc intervention is the integration of psychological approaches such as cognitive behavioural theory and motivational interviewing to address the “thinking trap” barriers to hypoglycaemia avoidance^23,40–42^. These are beliefs around hypoglycaemia that have been described by people with IAH and can reduce motivation to take the behavioural steps needed to prevent or reduce hypoglycaemia^23^. HARPdoc participants learned about the vicious cycle of impaired awareness, linking physical sensations (no symptoms), to feelings (I feel fine), to thoughts (I’ll keep going), to behaviours (don’t treat a hypo), hence missing the opportunity to correct the impaired response to falling blood glucose levels. Motivation is a key element in achieving behaviour change^43^. The HARPdoc educators were trained and supported to deliver the curriculum using non-judgemental language, to help participants reflect on their own ‘thinking traps’ without defensiveness and alter their behaviours in response to them.

The different impact of HARPdoc and BGAT on participant scores on the Attitudes to Awareness questionnaire suggests the former programme achieved its intended aim. Why this did not produce greater falls in the number of severe hypoglycaemic episodes compared to BGAT may have several explanations. It is possible that the “thinking traps” are not as important in sustaining problematic hypoglycaemia as we hypothesised. Other benefits from the courses might have had equal or greater impact: sharing experiences related to problematic hypoglycaemia, taking time out from daily life to focus on hypoglycaemia avoidance, revising information about the drivers of hypoglycaemia and how best to treat and avoid it, may all have led to a new prioritisation of hypoglycaemia avoidance in participants of both courses. It is also possible that the highly experienced diabetes educators delivering BGAT inadvertently used skills and knowledge that we had assumed were unique to HARPdoc. A study of the fidelity of treatment delivery is underway. However, there may be other explanations. Not all our participants entered the study possessing the unhelpful health beliefs that HARPdoc specifically addresses, as this was not part of our eligibility criteria, and those who did not have these thoughts may have done equally well with either intervention. This is something that can be explored in future.

There are two clinical implications for our work. Our cohort had extremely high baseline rates of severe hypoglycaemia, which they had been experiencing for a long time. Although most episodes were treated by family, friends and passers-by, there will have been an impact on health care resources, not just in terms of emergency care resource use but also health care professional time in having repeated, ineffective conversations. For ethical reasons we did not include an inactive control but the participants were largely fully engaged with specialist services (with neither HARPdoc nor BGAT available options) prior to recruitment and failing to achieve hypoglycaemia avoidance despite specialist support. BGAT has RCT data showing its ability both to reduce severe hypoglycaemia and improve hypoglycaemia awareness^29^; our participants had access to the recommended treatment pathway for managing hypoglycaemia risk in adults with T1D^2^ prior to recruitment and yet had long duration of problematic hypoglycaemia before entering the trial, so it is reasonable to ascribe some likely benefit to the interventions. This trial thus suggests that both BGAT and HARPdoc can lower severe hypoglycaemia rate, and improve hypoglycaemia awareness, in people with problematic hypoglycaemia who have already undertaken other evidence-based education programmes and in many cases also technology^3^.

The evidence for a beneficial impact of HARPdoc on emotional distress—both general and diabetes specific – in our trial comes from the secondary outcome data, and as such must be considered exploratory at present. The difference between the impact of HARPdoc and BGAT on scores for anxiety and depression remained significant after rigorous correction for multiple comparisons and the improvement across a few measures of mental health provides additional face validity. Although statistically the weakest of the three measures of mental health, the probable reduction in the PAID scores is of interest. PAID provides a measure of diabetes distress, which has been related to both IAH and to higher glycated haemoglobin^44^. Interestingly, a recent report of a successful intervention to improve both severe hypoglycaemia and glycated haemoglobin with closed loop insulin therapy did not reduce diabetes distress^45^. In the present study, mean baseline PAID scores were higher than in people not experiencing problematic hypoglycaemia^35^ and fell more after HARPdoc than BGAT, despite no greater reduction in problematic hypoglycaemia.. Similarly, scores for anxiety and depression, both significant problems for people living with diabetes often requiring treatment^46^, were high in our participants at baseline^39^ and appear to have fallen in the HARPdoc participants, to levels similar to those seen in people without problematic hypoglycaemia.^39^ An intervention that can reduce these burdens while also tackling problematic hypoglycaemia will be welcomed. Our findings imply that the depression and anxiety seen in IAH may not be caused by the hypoglycaemia directly, which is in line with our concept of IAH as a state of interoceptive unawareness. It is possible that HARPdoc’s therapeutic approach is helping relieve distress that is related to the hypoglycaemia through processes such as increased self-efficacy at dealing with hypoglycaemia, empowerment and self-reflection. The HARPdoc programme also addresses self-critical thinking which can drive depression and the catastrophic thinking style associated with anxiety, both of which may have generalised effect beyond the context of hypoglycaemia. It is worth noting that from the patients’ perspective, the time commitment of HARPdoc is not much greater than for BGAT. Meanwhile, IAH is only one example of resistance to change in asymptomatic medical conditions with potentially serious adverse outcomes. The HARPdoc model may have relevance outside insulin-driven hypoglycaemia.

Limitations of our study include absence of an inactive control arm, made necessary because participants were receiving state of the art care and BGAT was not available in its evidence-based English-language form; lack of diversity in the participants and the potential for the further development of diabetes technology during the trial period to have impacted on hypoglycaemia risk, although adjusting our models for rtCGM use and removing new users from the models make this last an unlikely major contributor. Our design does not allow us fully to explain the mechanisms of action by which severe hypoglycaemia was reduced in each arm of the trial. On-going research is needed further to explore the relative impacts of course content, educator skills and participant interaction as contributors to the outcomes. Using participant 12-month recall of severe hypoglycaemia might be considered a limitation, although it is the conventional method for assessing severe hypoglycaemia experience in trials and in clinical practice^47^. The use of anonymised questionnaires reduced risk of participant underreporting^48^ and accuracy of diagnosis was supported by the adjudication data, which suggested that participants were identifying episodes in line with international definitions^1^. Strengths of the study include the successful recruitment of the participant group in whom conventional therapies, including technological ones, have failed to eliminate the problems of severe hypoglycaemia and rigorous attention to detail in the trial conduct. The interventions were delivered in face-to-face format. Both interventions underwent formal evaluation of their implementation during the trial^49^, which will be reported subsequently. After completion of the trial interventions, we have begun to deliver HARPdoc courses using video conferencing. This delivery mechanism needs to be researched for both interventions.

We conclude that HARPdoc is not superior to BGAT in reducing severe hypoglycaemia in adults with T1D and problematic hypoglycaemia that has persisted despite otherwise optimised diabetes management. However, the data suggest that such psycho-educational interventions have positive impact to improve treatment-resistant hypoglycaemia and the psychotherapeutic approach of HARPdoc has the potential also to improve diabetes-specific and general distress. Such programmes should be available as adjuncts to technological solutions to minimise the burden of problematic hypoglycaemia in T1D.

## Methods

This research complies with the relevant regulations for the conduct of research in human volunteers. The protocol was approved by the London - Dulwich Research Ethics Committee, 16/LO/1992, for the UK’s Health Research Authority, and the Committee on Human Studies of the Joslin Diabetes Center (2016-32) and is posted on ClinicalTrials.gov (NCT02940873). The protocol is available from the authors and has been published as a paper.^27^ All participants gave written informed consent prior to any study procedure.

The population for which HARPdoc was designed is that of adults with T1D who continue to experience IAH and recurrent severe episodes, despite provision of an evidence-based hypoglycaemia minimisation pathway^2^, including at least structured education in flexible insulin therapy and availability of diabetes technologies that reduce severe hypoglycaemia^3^. The intervention being tested was HARPdoc itself, a manualised psycho-educational programme with a focus on modifying unhelpful thoughts related to hypoglycaemia, against the comparator, BGAT^28^, a psycho-educational programme which a focus on teaching new behaviours to avoid high and low blood glucose.

### Primary outcome

Rate of SH events (number of events over preceding year) measured using the 12 and 24-month anonymised SH recall forms^27^. Where anonymised data were missing, participants were asked for permission to use data reported in the equivalent open form, and to confirm that they agreed the data were a true reflection of their current experience.

### Secondary outcomes

These covered the type of hypoglycaemia event, the impact of courses on HbA1c and a range of psychological outcomes (cognitive, behavioural and emotional), listed in the supplementary data (Table S9).

### Study participants

We recruited adults with T1D who were continuing to report both IAH (as confirmed by a Gold and/or Clarke score of four or more^30,31^) and recurrent episodes of severe hypoglycaemia, despite having completed formal education in flexible insulin self-management. Severe hypoglycaemia was defined as an episode of low blood glucose requiring assistance of another person actively to administer corrective action, because of impaired cognitive function, or episodes of loss of consciousness or seizure^1^. Inclusion criteria included age 18 years or older; a clinical diagnosis of T1D of at least four years’ duration; experiencing problematic hypoglycaemia, defined as IAH and more than one severe hypoglycaemia in the last two years, with at least one occurring on their present treatment regimen; completion of structured education in flexible insulin therapy and on-going specialist care; current use of an appropriate (in the investigator’s estimation) multiple daily insulin injection regimen or CSII (insulin pump) therapy; willingness to comply with study design, including willingness and ability to perform home blood glucose testing up to four times a day routinely; ability to communicate in written and spoken English and give written informed consent. Exclusion criteria were type 2 diabetes; T1D with preserved awareness of hypoglycaemia; no previous structured education in flexible intensive insulin therapy; pregnancy; severe mental illness; cognitive impairment; diagnosed eating disorder; co-morbid medical disease contributing to hypoglycaemia (e.g. inadequately treated Addison’s disease or growth hormone deficiency or hypothyroidism; untreated coeliac disease; uncontrolled gastroparesis; end stage renal disease). Blood test result ruling out the co-morbidities could be taken from existing clinical data, provided the test had been carried out within 12 months, and during the period of the participant’s current hypoglycaemia problem. Participants were reimbursed for out-of-pocket expenses involved in attending study visits, either on presentation of receipts (UK) or as a fixed sum offered for each visit (USA).

The HARPdoc RCT was run contemporaneously in four sites, all specialist diabetes clinics providing secondary and tertiary care for adults with T1D and all offering structured education in flexible insulin self-management and access to diabetes technologies (insulin pumps, continuous glucose monitors and automated insulin delivery systems). Three centres were in the UK (one in London, comprising both King’s College Hospital and the Guy’s and St Thomas’ NHS Foundation Trusts); the Royal Bournemouth Hospital in Dorset and the Northern General Hospital in Sheffield; the fourth was the Joslin Diabetes Center in Boston, MA, in the US. Recruitment into initial courses fell short of the target of 16 participants per pair of courses and the London centre ran two additional courses.

### The intervention

HARPdoc is a six-week programme, delivered to small groups of participants by two diabetes educators (nurse or dietitian), trained and supported by a clinical psychologist. It comprises four full-day group sessions, in weeks one, two, three and six, with two individual consultations (face to face or remote) during weeks four and five. There are sessions for partners or other close family members on day six. The HARPdoc curriculum was piloted as “DAFNE-HART”^26^, after which the curriculum and participant guidebook were revised in line with feedback from participants and educators for use in this RCT. The curriculum revises and updates participants’ knowledge of hypoglycaemia, its manifestations and drivers and teaches participants how to maximise awareness of cues that hypoglycaemia is occurring. Its unique feature is that it uses specific psychological approaches, from motivational interviewing and cognitive behavioural theory. It directly addresses cognitive barriers to hypoglycaemia avoidance described by people with IAH^23,40^. These are referred to during the courses as “thinking traps” and made accessible to participants using visual metaphors: “The ostrich” with its head in the sand, reflecting minimisation of concern about hypoglycaemia—‘it’ll never happen to me’; “The over-sensitive smoke alarm”, reflecting fear of hyperglycaemia - ‘better to be low than high’ and “The Soldier”, reflecting normalisation of asymptomatic hypoglycaemia and soldiering on – ‘I don’t want to make a fuss’. The courses are designed for up to eight participants, and never less than four. If fewer than four participants were recruited to a HARPdoc course, additional patients not suitable for or recruited into the trial but thought by their clinicians to have a clinical indication for the intervention could be enroled. These patients and any data they provided are not included in the trial data.

### The comparator

BGAT is the only psycho-educational programme designed to help adults with T1D improve their glucose outcomes with trial data showing improved awareness of hypoglycaemia as well as reduced severe hypoglycaemia as outcomes^28^, although in a meta-analysis its impact on the latter was probably not greater than two other education packages, DPPT and HyPOS, the latter specifically designed to reduce severe hypoglycaemia^3^. BGAT is an eight-session manualised programme normally delivered by a single diabetes educator either one-to-one or to small groups of patients. It teaches participants how to predict and avoid high as well as low blood glucose, by increasing knowledge and understanding of drivers of glucose concentrations and focussing attention on feelings and experiences that may indicate an extreme value. For the purposes of this trial, the manual was reviewed and updated by the study BGAT educators, under the guidance of one of the psychology team which had created it, and one of the co-investigators in this trial (L.G.F.). The sessions, originally designed as eight two-hour group or one-to-one sessions, were re-planned as small group only to occupy the same time frame as the HARPdoc courses.

Each centre delivered courses in pairs, on the same dates. All participant facing literature (the HARPdoc participant work-book and the BGAT manual) was made available as hard copies, in both UK and US formats. Participant newsletters sent during the trial to keep participants informed of progress were likewise made available in each format.

### The educators

All the educators delivering either intervention were experienced diabetes specialist nurse or dietitian educators. Prior to starting recruitment, a minimum of three diabetes educators, two for HARPdoc and one for BGAT, were trained in the delivery of the courses in each centre. Initial training took place over two days. HARPdoc educators undertook a manualised two-day training in the delivery of HARPdoc, including training in motivational interviewing (MI) skills and an understanding of the cognitive behavioural theory (CBT) model of IAH with subsequent role-play and assessment including feedback, provided by the trial clinical psychologists. During course delivery, supervision was provided weekly, by the trial psychologist, to each centre to troubleshoot any clinical issues and reinforce key principles of the HARPdoc curriculum. The BGAT educators revised and updated the existing BGAT manual during the initial training and in two subsequent teleconferences and had access to the training psychologist for advice on demand during courses. No BGAT educator received HARPdoc training or exposure, or vice versa and care was taken throughout the course of the trial to avoid contamination.

### Randomisation

Eligible people expressing an interest were invited to a screening visit and if found to be eligible and willing were enroled and offered a face-to-face baseline data collection visit. Randomisation was provided by an independent service at the King’s Clinical Trials Unit (KCTU) and was carried out once a minimum of 11 participants had been recruited, no longer than one week prior to the start of courses. Randomisation Groups of 11–16 participants were randomised at the level of individual, using block randomisation with fixed block size of 2, stratified by country (UK/US) and use of technology (pumps and/or sensors; technology/no technology). Both the study subjects and educators delivering the treatment were unblind to treatment allocation. The trial statistician (L.P.) and senior statistician (IB) remained subgroup blind (only aware of coded trial arm memberships) until at the final stages of analysis, after all the data had been collected and cleaned.

### Data collection

Data collection took place at baseline and at 3, 6, 12, 18 and 24 months after randomisation, with more limited data collection at 3, 6 and 18 months. Participants also were asked to submit an anonymised form describing the event shortly after any severe hypoglycaemia, which was sent to two specialist diabetes physicians for adjudication, with a third adjudicator in the event of a disputed diagnosis. Monthly reminders were made.

HARPdoc follow-up was conducted in group format, with the educators present. At 3, 6 and 12 months the group visit included formal review of progress. For all participants, the full data set included an anonymised severe hypoglycaemia form in which participants recalled their experience of severe hypoglycaemia over the previous 12 and 24 months. These data formed the primary endpoint. A book of questionnaires including a further form collecting data on recalled severe hypoglycaemia experience over the previous 12 and 24 months; Gold and Clarke scores of hypoglycaemia awareness status, previous offers of interventions to reduce hypoglycaemia risk, current insulin regimen and method of glucose monitoring, the Attitudes to Awareness (A2A) covering the cognitive barriers to hypoglycaemia avoidance; the Hypoglycaemia Fear Survey (HSF-II) and other questionnaires as listed in Supplementary data Table S9 covering hypoglycaemia experience and impact of hypoglycaemia was completed by each participant prior to each of the baseline, 12 and 24-month visits and checked for completeness of data entry with the researcher or educator at the relevant visit. Anonymised forms were sent directly to the Trial Data Manager at the Trial Management site at King’s College London and entered directly into the trial’s electronic database (Elsevier InferMed MACRO Electronic Data Capture System) without clinical review. The KCTU created and supported the electronic database for the study, from templates created with the research team.

### Impact of covid pandemic

An online version of all the participant questionnaires was created in March 2020 using a Qualtrics platform (Qualtrics XM), checked for accuracy of transcription and made accessible to participants via an on-line link. Data collection visits were then conducted by telephone or using video conferencing, with face-to-face visits offered where regulations permitted, as the participant preferred. Blood samples were collected for central measurement of HbA1c at outstanding 24-month visits via home collection of capillary blood, using kits designed by the central laboratory and adopted after testing for durability of sample. Participant engagement was enhanced through additional editions of the trial participant newsletter.

### Sample size

As described in the published protocol^27^, enrolment of 96 participants was estimated to give 90% power at 2.5% level of significance.

A base rate of 10 SH episodes per year was assumed for this study, using data from both the DAFNE-HART pilot^26^ and the HypoCOMPaSS trial^50^.

The mean rate of SH following DAFNE-HART was 0.5 per subject year at 12 months. The dropout rate was 5%. For present purposes, we considered also a much more conservative estimate of HARPdoc success, namely two SH events per person per year. This is between the average rates reported in the studies quoted above.

For BGAT, the literature reports a range of outcomes, SH rates falling by one-third to nearly 80% in one study in the Netherlands. All participants in the present RCT have completed structured education in flexible intensive insulin therapy so we investigated a series of more conservative estimate of the impact of BGAT. The mean rate of SH following treatment of BGAT ranged from 3.8 (the mean of 3 published studies) to 6.9 (the maximum) per subject year at 12 months. Difference of 3.8 was used in the final sample size calculation.

We inflated the sample size to take account of within-group correlation and adjusted for therapist group, for which the intra-class correlation was estimated as 0.02^51^. It was envisaged that therapy groups will have between 6 and 8 patients giving a design effect of 1 + 0.02 (8–1) = 1.14. We adjusted for multiple comparison with the use of Bonferroni Correction in two endpoints (12 and 24 months) (corrected alpha = 0.025%, two sided).

### Biochemical analyses

At baseline, 12 and 24 months blood samples were sent to the central laboratory at ViaPath at King’s College Hospital London for measurement of HbA1c using High-Pressure Liquid Chromatography (Premier 9210 analyser, Menarini, Italy). The same laboratory and methods were used for samples collected and sent in from home during Covid restrictions.

### Statistical analyses

The statistical analysis plan (HARPdoc statistical analysis plan v1.11 16062020) was developed by the trial team and approved by the trial steering committee before database lock. Baseline data were summarised and as per the Statistical Analysis Plan, no statistical significance tests were carried out at baseline as randomisation ensures that any imbalance over all measured and unmeasured baseline characteristics is due to chance.

The main analysis was an ITT analysis using all available follow-up data from all randomised participants. The statisticians were blinded to treatment allocation during the ITT analyses and were unblinded prior to any additional analyses such as the PP analysis. For the primary analysis, the outcome was the count of the number of SH events over the preceding year per participant at 12 and 24 months post randomisation, taken from the anonymised recall forms. Where anonymised forms had not been submitted within the appropriate time window, participants were asked for permission to use the open data collected at the scheduled visit if they agreed the open data were an accurate reflection of their experience. Initially, a complete case analysis was performed under a missing at random assumption (MAR) where explanatory variables could predict the missing values in the respective outcome variable. These models drop any participant who does not provide outcome data at the follow-up time point.

Because the primary outcome data were an “overdispersed” count outcome (12 months: mean = 8.6, SD = 27.4; 24 months: mean = 4.2, SD = 20.2), and the overdispersion parameter differed significantly from zero (p < 0.0001), negative Binomial regression was used to model the hypoglycaemia data. To compare the randomised groups at 12 and 24 months’ follow-up adjusting for baseline number of SH events in the year preceding randomisation and accounting for hierarchical clustering of patients at the level of therapy group, we fit a three-level random intercept Negative Binomial model including observations from 12 and 24 months at level 1, individuals at level 2 and therapy groups at level 3. The model included the count of SH up to 12 and between 12 and 24 months as the outcome variables with treatment group, baseline rate of SH and stratifiers (country and use of technology) as explanatory variables. A time by treatment interaction was included to allow the effect to differ at each time point. Both complete case (under a MAR assumption) and an analysis adjusted for missing data biases using MICE are presented. A significance level of 2.5% (two sided) to account for the comparison being performed at two time points (12 and 24 months) was used.

A similar methodology was used for secondary outcomes, with a random intercept negative binomial model for all hypoglycaemia count outcomes. A random intercept linear regression model was used for all other outcomes, except for the number of participants in whom HbA1c did not rise by 0.3% or more, for which a random intercept logistic regression model was used. Normal outcomes used an identity link function with estimates presented as a mean difference, binary variables used a logit link function with estimates presented as an odds ratio, and count variables used a log link function with estimates presented as an incident rate ratio. Data from 3, 6, and 18 month follow-up visits were available for Gold and Clarke scores which were also included as dependent variables in the models. Due to minimal events of severe hypoglycaemia with hospital admission (one night or more) (12 months: 0 events; 24 months: 3 events), we were only able to summarise this secondary outcome and unable to model and formally assess any differences between trial arms. We used a significance level of 5% (two sided) for secondary outcome analyses, which becomes 2.5% after Bonferonni correction for multiple comparisons. Pro-rating (where any missing items are replaced by the mean score of the non-missing items) was used in the Clarke, A2A, PAID and HADS scales where less than 20% of items were missing. Mean item scores for HFS-II and HAS scales were used when less than 25% of items were missing, in accordance with guidance from the literature and the authors of the published scales.

Using the protocolised definition of non-completion of therapy (“non-compliance”), attendance at least three full-day sessions for HARPdoc to include day 3 and also at least one of the one-to one sessions, completion was found to predict missingness of primary outcome data (chi-squared (1) = 9.82, *p* = 0.0017). MICE was used to produce inferences valid under a MAR that allowed observed non-completion to drive missingness. Each outcome (primary and secondary) had a separate MI model, where all time points including baseline (where missingness was observed) were imputed in the same model. Independent variables used in the imputation model were the intervention, the stratifiers, the course, any baseline predictors of missingness and completion. By univariable logistic regression the only baseline measure associated with missingness of the primary outcome at a liberal 10% test level was gender, for which data collection was complete, which was included in the multiple imputation (MI) model. For both primary and secondary analyses, both complete case (under a MAR and an analysis adjusted for missing data biases using MICE) were undertaken.

A PP analysis was also conducted in which the primary outcome was compared between groups, removing the data of those who had not complied with the intervention by: delayed start of their course by more than two months post randomisation; later being found to have an exclusion criterion that had been missed, including new pregnancy; the participant undertaking islet or pancreas transplant or whose outcome data were collected outside the visit window. A pre-specified subgroup analysis of participants in the trial with low worry and low behaviour, defined as participants who score less than 0.92 on the worry subscale or 1.85 on the behaviour subscale of the Hypoglycaemia Fear Survey II, was also conducted with the inclusion of an interaction term with the treatment arm (HARPdoc vs BGAT). Results of each subgroup were presented, alongside a test for an interaction. The study has not been powered for subgroup effects or interactions so therefore this is an exploratory analysis only.

An additional subgroup analysis also tested for any effects COVID-19 may have had on the trial with the inclusion of an interaction term with the treatment arm (HARPdoc vs BGAT). There were 2 subgroups, pre-lockdown (pre-March 2020) and post lockdown (post March 2020). The subgroup analysis was applied to the primary outcome at 24 months only, as the 12 months outcome data was unaffected by Covid-19. The HARPdoc v BGAT treatment effect was estimated in each of the subgroups.

Regression assumptions were checked for all outcomes, and imputed datasets were checked to make sure they had a similar distribution to the observed data.

### Reporting summary

Further information on research design is available in the Nature Research Reporting Summary linked to this article.

## Supplementary information

Supplementary Information

Description of Additional Supplementary Information

Reporting Summary

## Data Availability

This work, started in 2017, has no protocolised provision for data sharing. Exploratory data analyses by the investigators are on-going. Individual de-identified participant data are available by application to the chief investigator (S.A.A.) and/or senior author I.B. from bona fide researchers interested in undertaking meta-analyses or on-going research, ordinarily with one or more of the original study PIs as collaborator or sponsor, in line with our institutional policies, from the date of publication for five years. Data files shared in this way may not then be shared with others. The protocol is available from the authors and has been published as a paper^27^, as outlined in the “Methods”. Source data are provided with this paper.

## Source data

Source Data

## Code availability

Full computer code was created in STATA 15.0 (tataCorp. 2017. Stata Statistical Software: Release 15. College Station, TX: StataCorp LLC.) and is available from the authors (S.A.A. or I.B.) on request.
