# Supplementary material for: A parallel randomised controlled trial of the Hypoglycaemia Awareness Restoration Programme for adults with type 1 diabetes and problematic hypoglycaemia despite optimised self-care (HARPdoc)
Source: Nat Commun. 2022 Apr 28;13:2229. doi: 10.1038/s41467-022-29488-x (PMC9050729; doi:10.1038/s41467-022-29488-x)
Supplement: Supplementary file 1 — Supplementary Information [file 41467_2022_29488_MOESM1_ESM.pdf]

Supplementary Table S1: HARPdoc RCT: Randomisation by site and arm. Summary statistics are presented using n (%).

Site, n(%)	Country	HARPdoc	BGAT	Overall
King's Health Partners	UK	24 (49.0)	21 (42.0)	45 (45.4)
Sheffield Teaching Hospitals NHS Foundation Trust	UK	10 (20.4)	10 (20.0)	20 (20.2)
Royal Bournemouth General Hospital	UK	7 (14.3)	10 (20.0)	17 (17.2)
Joslin Diabetes Center, Boston	USA	8 (16.3)	9 (18.0)	17 (17.2)
Total		49	50	99

Supplementary Table S2. Withdrawals, losses to follow-up and protocol violations or deviations from the allocated treatment, by treatment arm. Withdrawals are presented as totals and in cumulative fashion based on the timing of and reason for withdrawal. Summary statistics are presented using n(%).

	HARPdoc n (%)	BGAT n(%)
Withdrawals		
N	49	50
Withdrew from trial, total	6 (12.2)	6 (12.0)
prior to 3 months	3 (6.1)	2 (4.0)
prior to 12 months	4 (8.2)	5 (10.0)
prior to 24 months	6 (12.2)	6 (12.0)
Reasons for withdrawal		
No longer willing to participate	3 (50.0)	2 (33.3)
Time demands too great	1 (16.7)	0 (0.0)
Lost to follow-up	1 (16.7)	2 (33.3)
Death	1 (16.7)	2 (33.3)
Protocol violations		
a. Did not receive allocated treatment as per protocol¥	5 (10.2)	9 (18.0)
b. Delayed undertaking intervention by more than 2 months after randomisation	1 (2.0)	3 (6.0)
c. Found to have an exclusion criterion	3 (6.1)	2 (4.0)
d. Islet transplant during the trial	0 (0.0)	1 (2.0)
e. Primary outcome outside of visit window at 12 months	5 (10.2)	4 (8.0)
f. Primary outcome is outside of visit window at 24 months	3 (6.1)	4 (8.0)
Removed from Per Protocol analysis*– n(%)	9 (18.4)	14 (28.0)

¥ Attendance of at least first 3 days of allocated treatment (HARPdoc or BGAT) plus at least one 1:1 session for HARPdoc considered as 'received allocated treatment'.

* Removed due to meeting any of criteria (a) to (d) or meeting criteria both (e) and (f) resulting in all primary outcome data being removed from the model. Deviations (b) to (d) could not be derived from the database and were provided by the study team with a list of participant pin numbers.

Supplementary Table S3. Pre-specified subgroup analysis showing impact of intervention on primary outcome (12 month recall of severe hypoglycaemia (SH) events using anonymised reporting) amongst HFS-II worry and behaviour subgroups. Effect estimates (Incidence Rate Ratios (IRR) from a three-level random intercept negative binomial model) with 95% CI are presented. Significance level of 2.5% (two-sided). N = number of participants with data

HSF-II Worry Subscale score	< 0.92		> 0.92	
	Estimated treatment effect	P	Estimated treatment effect	P
SH rate<br>12 months	N=70<br>1.13 [0.12, 10.71]	0.92	N=17<br>1.18 [0.44, 3.17]	0.74
SH rate<br>24 months	N=67<br>0.76 [0.06, 9.30]	0.83	N=16<br>1.40 [0.48, 4.11]	0.54
HSF-II Behaviour Subscale score	<1.85		>1.85	
	Estimated treatment effect	P-value	Estimated treatment effect	P-value
SH rate<br>12 months	N=65<br>1.49 [0.52, 4.25]	0.46	N=22<br>0.80 [0.15, 4.38]	0.80
SH rate<br>24 months	N=63<br>1.11 [0.24, 5.15]	0.89	N=20<br>0.33 [0.05, 2.07]	0.24

Supplementary Table S4a: Secondary analysis adjusted for missing data biases using MICE: Glycaemic variables. Differences in rates of severe hypoglycaemia (SH) episodes are assessed using a three-level random intercept negative binomial regression analysis and estimated treatment effects presented as an incidence rate ratio (IRR). Other outcomes are analysed using a three-level random intercept linear (mean difference), or logistic regression (Odds Ratio (OR)) model. All estimates are presented with a 95% CI and P<0.05 (two-sided) are considered significant. N = number with data

Secondary Outcome	N	Estimated treatment effect	(95% CI)	p-value
SH rate where loss of consciousness or seizure 12 months	99	0.79	(0.27, 2.31)	0.67
SH rate where loss of consciousness or seizure 24 months	99	1.47	(0.46, 4.73)	0.52
SH rate where glucagon or IV glucose given 12 months	99	0.71	(0.25, 2.00)	0.52
SH rate where glucagon or IV glucose given 24 months	99	1.74	(0.54, 5.54)	0.35
SH rate with ambulance call out 12 months	99	0.71	(0.21, 2.34)	0.57
SH rate with ambulance call out 24 months	99	2.24	(0.66, 7.56)	0.19
SH rate with A and E attendance 12 months	99	2.10	(0.24, 18.04)	0.50
SH rate with A and E attendance 24 months	99	1.02	(0.09, 11.88)	0.99
SH rate with hospital admissions (one night or more) 12 months	99	Low number of events	Unable to analyse	
SH rate with hospital admissions (one night or more) 24 months	99	Low number of events	Unable to analyse	
Rate of moderate hypoglycaemia episodes 12 months	99	1.12	(0.58, 2.14)	0.74
Rate of moderate hypoglycaemia episodes 24 months	99	0.89	(0.46, 1.72)	0.74
Central HbA1c 12 months	99	-0.05	(-0.31, 0.22)	0.72
Central HbA1c 24 months	99	-0.06	(-0.36, 0.25)	0.71
Proportion of people in whom HbA1c did not rise by more than 0.3% 12 months	99	OR: 1.28	(0.28, 5.95)	0.75
Proportion of people in whom HbA1c did not rise by more than 0.3% 24 months	99	OR: 4.06	(0.59, 27.98)	0.16
Hypoglycaemia Awareness Status				
Gold score 12 months	99	-0.41	(-0.98, 0.17)	0.17
Gold score 24 months	99	-0.24	(-0.85, 0.38)	0.45
Clarke score 12 months	99	-0.10	(-0.66, 0.47)	0.74
Clarke score 24 months	99	0.23	(-0.36, 0.81)	0.45

Supplementary Table 4b: Secondary analysis adjusted for missing data biases using MICE: Cognitive, emotional and behavioural variables. Differences are assessed using a three-level random intercept linear regression analysis and estimated treatment effects presented as a mean difference with a 95% CI. P<0.05 (two-sided) are considered significant. N = number with data

Secondary Outcome	N	Estimated treatment effect	(95% CI)	p-value
Attitudes to Awareness (A2A) Questionnaire				
A2A (hyperglycaemia avoidance prioritised score) 12 months	99	-0.69	(-1.58, 0.19)	0.13
A2A (hyperglycaemia avoidance prioritised score) 24 months	99	-0.98	(-1.90, -0.07)	0.04
A2A (hypoglycaemia concern minimised score) 12 months	99	-0.22	(-0.82, 0.43)	0.54
A2A (hypoglycaemia concern minimised score) 24 months	99	-0.60	(-1.28, 0.07)	0.08
A2A (asymptomatic hypoglycaemia normalized) 12 months	99	-0.50	(-1.15, 0.14)	0.13
A2A (asymptomatic hypoglycaemia normalized) 24 months	99	-0.36	(-0.99, 0.26)	0.26
A2A total 12 months	99	-1.46	(-2.89, -0.02)	0.05
A2A total 24 months	99	-2.15	(-3.60, -0.69)	0.01
Hypoglycaemia Fear Score (HSF-II)				
HFS-II behaviour score 12 months	99	-0.18	(-0.40, 0.04)	0.11
HFS-II behaviour score 24 months	99	-0.04	(-0.27, 0.19)	0.74
HFS-II worry score 12 months	99	-0.01	(-0.25, 0.24)	0.98
HFS-II worry score 24 months	99	0.07	(-0.20, 0.33)	0.63
HFS total score 12 months	99	-0.08	(-0.27, 0.12)	0.45
HFS total score 24 months	99	0.01	(-0.21, 0.23)	0.91
HAS behaviour score 12 months	99	-0.14	(-0.33, 0.05)	0.15
HAS behaviour score 24 months	99	-0.03	(-0.23, 0.17)	0.77
HAS worry score 12 months	99	-0.18	(-0.40, 0.03)	0.10
HAS worry score 24 months	99	-0.21	(-0.44, 0.01)	0.07
HAS total score 12 months	99	-0.16	(-0.34, 0.03)	0.10
HAS total score 24 months	99	-0.11	(-0.30, 0.07)	0.23
Measures of mental health				
Diabetes distress (Problem Areas in Diabetes)				
PAID total score 12 months	99	-5.53	(-11.36, 0.30)	0.06
PAID total score 24 months	99	-5.84	(-11.79, 0.11)	0.05
Hospital Anxiety and depression score (HADS)				
HADS anxiety score 12 months	99	-1.71	(-3.11, -0.30)	0.02
HADS anxiety score 24 months	99	-1.87	(-3.34, -0.41)	0.01
HADS depression score 12 months	99	-1.82	(-3.25, -0.38)	0.01
HADS depression score 24 months	99	-1.98	(-3.49, -0.47)	0.01

Supplementary Table S5: Uptake of diabetes technology during the course of the trial by intervention group. n = number of participants providing data. Summary statistics are presented using n(%), and "N=" represents number of participants providing data

	HARPdoc 12 months	BGAT 12 months	HARPdoc 24 months	BGAT 24 months
Total number in group	49	50	49	50
Uptake of pump, number (%) new users reported at time point	N=42<br>1 (2.4)	N=43<br>1 (2.3)	N=37<br>1 (2.7)	N=40<br>1 (2.5)
Uptake of pump with automated suspend feature, number (%) new users reported at time point	N=41<br>2 (4.9)	N=40<br>1 (2.5)	N=37<br>4 (10.8)	N=39<br>1 (2.6)
Uptake of CGM including Flash with Miao Miao, number (%) new users reported at time point	N=42<br>3 (7.1)	N=43<br>3 (7.0)	N=37<br>5 (13.5)	N=40<br>9 (22.5)
Uptake of bolus advisor, number (%) new users reported at time point	N=41<br>4 (9.8)	N=42<br>7 (16.7)	N=37<br>3 (8.1)	N=40<br>5 (12.5)
Uptake of pump with or without automated suspend feature, CGM (including Flash with Miao Miao), number (%) new users reported at time point	N=42<br>9 (21.4)	N=43<br>12 (27.9)	N=37<br>10 (27.0)	N=40<br>11 (27.5)

Supplementary Table S6: Adverse events by from the trial period for all trial participants from the trial period for all trial participants arm (N=99). N = number with data, n = number of people with events

		HARPdoc N=49	BGAT N=50	Total N=99
Serious Adverse Events	n, events (people)	15 (9)	6 (5)	21 (14)
Body system code (number of events), n(%)	Metabolic - DKA	1 (6.7)	0 (0.0)	1 (4.8)
	Musculo-skeletal - falls or fractures	0 (0.0)	1 (16.7)	1 (4.8)
	Mental Heath	1 (6.7)	1 (16.7)	2 (9.5)
	Cardiovascular	2(13.3)	4(66.7)	6 (28.6)
	Other	11(17.3)	0(0)	11(52.4)
Adverse Events	n, people (events)	25 (15)	20 (12)	45 (27)
Body system code (number of events), n(%)	Metabolic – DKA	1 (4.0)	0 (0.0)	1 (2.2)
	Musculo-skeletal - falls or fractures	4 (16.0)	2 (10.0)	6 (13.3)
	Mental Heath	3 (12.0)	0 (0.0)	3 (6.7)
	Cardiovascular	0 (0)	1 (5.0)	1 (2.2)
	Other	17 (68.0)	17 (85.0)	34 (77.8)

Supplementary table S7. Impact of Covid pandemic on primary outcome data. Pre-specified subgroup analysis showing impact of intervention on primary outcome (12 month recall of severe hypoglycaemia (SH) events using anonymised reporting) amongst pre/post Covid-19 subgroups. Effect estimates (Incidence Rate Ratios (IRR) from a three-level random intercept negative binomial model) with 95% CI are presented from the trial period for all trial participants. SH = severe hypoglycaemia

	N	Estimated treatment effect	p-value	n	Estimated treatment effect	p-value
SH rate 12 months	26	0.67 [0.13, 3.43]	0.63	61	1.66 [0.56, 4.86]	0.36
SH rate 24 months	25	0.67 [0.11, 3.97]	0.66	58	2.04 [0.39, 10.58]	0.40

Supplementary table S8a: Comparison of 24 month data in HARPdoc RCT collected before and during Covid-19 restrictions: Glycaemic outcomes. Summary statistics are presented and (N=) represents available data; n= number; SH = severe hypoglycaemia

Outcome		Pre-covid	During covid
Total number of participants		25	58
SH episodes in past 12 months	Median (IQR)	N=25<br>0.0 (0.0-2.0)	N=58<br>0.0 (0.0-2.0)
Loss of consciousness/ seizure	Median (IQR)	N=16<br>0.0 (0.0-0.0)	N=53<br>0.0 (0.0-1.0)
Glucagon or IV glucose	Median (IQR)	N=16<br>0.0 (0.0-0.0)	N=53<br>0.0 (0.0-1.0)
Ambulance call out	Median (IQR)	N=16<br>0.0 (0.0-0.0)	N=53<br>0.0 (0.0-0.0)
A and E attendances	Median (IQR)	N=16<br>0.0 (0.0-0.0)	N=53<br>0.0 (0.0-0.0)
Hospital admissions	Median (IQR)	N=16<br>0.0 (0.0-0.0)	N=53<br>0.0 (0.0-0.0)
Moderate hypoglycaemia episodes in the past 4 weeks	Median (IQR)	N=23<br>6.0 (1.0-10.0)	N=47<br>3.0 (1.0-9.0)
HbA1c (%)	Mean ± SD	N=24<br>7.6 ± 1.2	N=45<br>7.3 ± 1.1
HbA1c rise ≤ 0.3%	n (%)	N=24<br>14 (58.3)	N=45<br>36 (80.0)
Hypoglycaemia awareness status			
Gold score	Mean ± SD	N=24<br>3.6 ± 1.5	N=55<br>4.0 ± 1.9
Gold score	number (%) scoring ≤ 3	14 (58.3)	23 (41.8)
Clarke score	Mean ± SD	N=25<br>3.9 ± 1.6	N=55<br>4.1 ± 1.6
Clarke score	number (%) scoring ≤ 3	9 (36.0)	19 (34.5)

Supplementary table S8b: Comparison of 24 month data in HARPdoc RCT collected before and during Covid-19 restrictions: Cognitive, emotional and mental health measures outcomes. Summary statistics are presented and (N=) represents available data.

Outcome	Pre-covid	During covid
Total number of participants	30	69
Attitudes to Awareness Questionniare (A2A)		
Hyperglycaemia Avoidance Prioritised<br>Mean ± SD	N=25<br>5.0 ± 1.9	N=52<br>4.2 ± 2.4
Hypoglycaemia Concern Minimised<br>Mean ± SD	N=25<br>2.4 ± 1.8	n=52<br>2.3 ± 1.9
Asymptomatic Hypoglycaemia Normalised<br>Mean ± SD	N=25<br>1.0 ± 1.2	N=52<br>1.3 ± 1.8
A2A total score<br>Mean ± SD	N=25<br>8.4 ± 2.4	N=52<br>7.9 ± 4.4
Hypoglycaemia Fear Survey, HFS-II		
HFS-II Behaviour subscale score<br>Mean ± SD	N=24<br>1.3 ± 0.9	N=52<br>1.4 ± 0.8
HSF-II Worry subscale score<br>Mean ± SD	N=24<br>1.3 ± 1.1	N=52<br>1.2 ± 0.9
HFS-II Total score<br>Mean ± SD	N=24<br>1.3 ± 0.9	N=52<br>1.3 ± 0.8
Hyperglycaemia Avoidance Survey (HAS)		
HAS Behaviour subscale<br>Mean (sd)	N=24<br>1.5 ± 0.4	N=51<br>1.5 ± 0.6
HAS Worry Subscale<br>Mean ± SD	N=25<br>1.8 ± 0.6	N=52<br>1.8 ± 0.7
HAS total score<br>Mean ± SD	N=24<br>1.7 ± 0.5	N=52<br>1.6 ± 0.5
Problem Areas In Diabetes (PAID)		
Total score<br>Mean ± SD	N=25<br>25.9 ± 20.4	N=52<br>25.0 ± 19.4
Hospital Anxiety and Depression Score		
HADS Anxiety<br>Mean ± SD	N=25<br>7.0 ± 4.8	N=52<br>6.5 ± 5.0
Score 8 or more – n(%)	11 (44.0)	20 (38.5)
HADS Depression<br>Mean ± SD	N=25<br>6.0 ± 4.8	N=52<br>5.6 ± 5.0
Score 8 or more n(%)	7 (28.0)	18 (34.6)

Supplementary Table S9. Secondary outcomes considered in the HARPdoc RCT. All data were collected at baseline, and at 12 and 24 months post-randomisation, with additional data on Gold and Clarke scores at 3, 6 and 18 months, by protocol.

Glycaemic outcomes		
Severe hypoglycaemia recalled over 12 months	Data source	Reference
Loss of consciousness or seizure	Anonymous	24
Administration of glucagon or IV glucose	Anonymous	"
Ambulance call outs	Anonymous	"
A and E attendances	Anonymous	"
Hospital admissions (≥ 1 night)	Anonymous	"
Moderate hypoglycaemia recalled over 4 weeks	Open	"
HbA1c (%)	Central laboratory	"
Participants with HbA1c rise ≤0.3%	Central laboratory	"
Awareness of Hypoglycaemia		
Gold Score Mean ± SD	Open	30
Gold Score No. of people scoring ≤3	Open	"
Clarke Score Mean ± SD	Open	31
Clarke Score No. of people scoring ≤3	Open	"
Endorsing cognitive barriers:<br>Attitudes to Awareness (A2) questionnaire		40
A2A Factor: Hyperglycaemia Avoidance Prioritised mean±SD	Questionnaire Booklet	"
A2A Factor: Hypoglycaemia Concern Minimised mean±SD	Questionnaire Booklet	"
A2A: Asymptomatic Hypoglycaemia Normalised mean ± SD	Questionnaire Booklet	"
A2A Total score mean±SD	Questionnaire Booklet	"
Fear of hypoglycaemia: Hypoglycaemia Fear Survey (HFS-II)		
HFS-II Worry Subscales scores mean±SD	Questionnaire Booklet	47
HFS-II Behaviour Subscale scores mean±SD	Questionnaire Booklet	"
HFS-II Total scores mean±SD	Questionnaire Booklet	"
Fear of Hyperglycaemia: Hyperglycaemia Avoidance Survey		
HAS Worry subscales scores mean±SD	Questionnaire Booklet	49
HAS Behaviour subscale scores mean±SD	Questionnaire Booklet	"
HAS Total score mean±SD	Questionnaire Booklet	"
Diabetes distress (PAID)		
PAID Total score mean ±SD	Questionnaire Booklet	32
HADS-A Mean ± SD	Questionnaire Booklet	33
HADS A No of people scoring ≤3	Questionnaire Booklet	"
HADS-D Mean ± SD	Questionnaire Booklet	"
HADS-D No of people scoring ≤3	Questionnaire Booklet	"

Supplementary Table S10. Members of the HARPdoc Research Group

Site	Names and Roles
London, UK	Stephanie Amiel (Chief Investigator); Mustabshira Qayyum (Trial Manager); Sean Haywood (Data Manager); Dulmini Kariyawasam (PI); Peter Jacob (research fellow); Pratik Choudhary (co-investigator); Nicole de Zoya (clinical psychologist, HARPdoc); Helen Rogers (Nurse consultant, lead educator); Emma L Smith (trial psychologist); Sarah Newell (dietitian educator); Victoria Francis (dietitian educator); Marietta Stadler (researcher); Rory Maclean (academic trainee); Karolina Bogdanowicz (Trial originator); Linda East, Sophia Coker, Shray Choudhary and Natalie Zaremba (centre administrative support).
Sheffield, UK	Simon Heller (PI); Val Gordon (diabetes nurse educator); Jill Rimmell (diabetes educator); Susan Hudson (diabetes educator); Sharon Caunt (research administration); Chloe Husband (research administrator).
Bournemouth, UK	Augustin Brooks (PI); Tanith Changuion (research administrator); Melanie Wiess (diabetes educator); Elsa Redfearn (diabetes educator); Emma Jenkins (diabetes educator); Jacqueline Ryder (diabetes educator).
Joslin Diabetes Center, USA	Elena Toschi (PI); Astrid Atakov-Castillo (research administrator); Lois Maurer (diabetes educator); Nicole Patience (diabetes educator); Monet Bland (diabetes educator).
University of Virginia, USA	Linda Gonder-Frederick (clinical psychologist, BGAT).
Institute of Psychiatry, Psychology and Neuroscience, London, UK	Kimberley Goldsmith (senior statistician); Ioannis Bakolis (lead statistician); Laura Potts (trial statistician).
Implementation Science, London, UK	Nick Sevdalis (PI); Tayana Soukup (researcher)
PPI group	Mike Kendall (chair), Vicky Ruszala; Arthur Durrant; Melanie Stephenson-Gray; Lis Warren; Richard Lane OBE.
