# Supplementary material for: A parallel randomised controlled trial of the Hypoglycaemia Awareness Restoration Programme for adults with type 1 diabetes and problematic hypoglycaemia despite optimised self-care (HARPdoc)
Source: Nat Commun. 2022 Apr 28;13:2229. doi: 10.1038/s41467-022-29488-x (PMC9050729; doi:10.1038/s41467-022-29488-x)
Supplement: Supplementary file 2 — Description of Additional Supplementary Information [file 41467_2022_29488_MOESM2_ESM.pdf]

Description of Additional Supplementary Information

NCOMMS-21-46984T: Inventory of supporting information

- Supplementary data – pdf
- Cover letter to editor
- Response to reviewers
- Author checklist
- Data files for figs 2 and 3
- CONSORT checklist for abstracts of clinical trials
- CONSORT checklist for manuscript
- Editorial policy checklist
- Reporting summary
