# Supplementary material for: A parallel randomised controlled trial of the Hypoglycaemia Awareness Restoration Programme for adults with type 1 diabetes and problematic hypoglycaemia despite optimised self-care (HARPdoc)
Source: Nat Commun. 2022 Apr 28;13:2229. doi: 10.1038/s41467-022-29488-x (PMC9050729; doi:10.1038/s41467-022-29488-x)
Supplement: Supplementary file 3 — Reporting Summary [file 41467_2022_29488_MOESM3_ESM.pdf]

nature portfolio

Corresponding author(s): Stephanie A Amiel

Last updated by author(s): Feb 11, 2022

# Reporting Summary

Nature Portfolio wishes to improve the reproducibility of the work that we publish. This form provides structure for consistency and transparency in reporting. For further information on Nature Portfolio policies, see our Editorial Policies and the Editorial Policy Checklist.

## Statistics

For all statistical analyses, confirm that the following items are present in the figure legend, table legend, main text, or Methods section.

| n/a | Confirmed | |
|---|---|---|
| ☐ | ☒ | The exact sample size (*n*) for each experimental group/condition, given as a discrete number and unit of measurement |
| ☐ | ☒ | A statement on whether measurements were taken from distinct samples or whether the same sample was measured repeatedly |
| ☐ | ☒ | The statistical test(s) used AND whether they are one- or two-sided<br>*Only common tests should be described solely by name; describe more complex techniques in the Methods section.* |
| ☐ | ☒ | A description of all covariates tested |
| ☐ | ☒ | A description of any assumptions or corrections, such as tests of normality and adjustment for multiple comparisons |
| ☐ | ☒ | A full description of the statistical parameters including central tendency (e.g. means) or other basic estimates (e.g. regression coefficient) AND variation (e.g. standard deviation) or associated estimates of uncertainty (e.g. confidence intervals) |
| ☐ | ☒ | For null hypothesis testing, the test statistic (e.g. *F*, *t*, *r*) with confidence intervals, effect sizes, degrees of freedom and *P* value noted<br>*Give P values as exact values whenever suitable.* |
| ☒ | ☐ | For Bayesian analysis, information on the choice of priors and Markov chain Monte Carlo settings |
| ☐ | ☒ | For hierarchical and complex designs, identification of the appropriate level for tests and full reporting of outcomes |
| ☐ | ☒ | Estimates of effect sizes (e.g. Cohen's *d*, Pearson's *r*), indicating how they were calculated |

*Our web collection on statistics for biologists contains articles on many of the points above.*

## Software and code

Policy information about availability of computer code

| | |
|---|---|
| Data collection | Data were entered into and stored in the electronic data base system of the King's Clinical Trial Unit, the Elsevier-InferMed MACRO Electronic Data Capture system. A study specific data base was created for the trial by members of the KCTU. |
| Data analysis | Stata 15.0 (StataCorp. 2017. Stata Statistical Software: Release 15. College Station, TX: StataCorp LLC.) was used for data description and the main inferential analysis. Full computer code which was created in STATA 15.0 is available from the authors (SAA or IB) on request. |

For manuscripts utilizing custom algorithms or software that are central to the research but not yet described in published literature, software must be made available to editors and reviewers. We strongly encourage code deposition in a community repository (e.g. GitHub). See the Nature Portfolio guidelines for submitting code & software for further information.

## Data

Policy information about availability of data

All manuscripts must include a data availability statement. This statement should provide the following information, where applicable:

- Accession codes, unique identifiers, or web links for publicly available datasets
- A description of any restrictions on data availability
- For clinical datasets or third party data, please ensure that the statement adheres to our policy

Following discussion with the editor, we have retained the procedure of making data available through the authors (SAA or IB) which we understand will comply with your policies. Our trial was started in 2017 and pre-dated the ICMJE guidelines, so there is no provision for data sharing in the original protocol (although participants did consent to anonymised data sharing with other researchers, Because there is no original provision was set up in the protocol and also because further exploratory analyses are pending, we have opted to offer data sharing on request. We are  including the statement "Anonymised trial data are available by application to the chief investigator (SAA) and/or senior author IB from bona fide researchers interested in undertaking meta-analyses or on-going research,

ordinarily with one or more of the original study PIs as collaborator or sponsor, in line with our institutional policies. Data files shared in this way may not then be shared with others." We believe this provides the information requested of such a plan which allows us to share de-identified patient level data with other researchers. We would expect them to approach us with one of the PIs of the existing study either as collaborator or sponsor and will then put in place a sharing agreement in line with our institutions' policies. We are not applying a time restriction. We are happy to edit this text and our plans if this does not meet your policies.

# Field-specific reporting

Please select the one below that is the best fit for your research. If you are not sure, read the appropriate sections before making your selection.

☒ Life sciences ☐ Behavioural & social sciences ☐ Ecological, evolutionary & environmental sciences

For a reference copy of the document with all sections, see nature.com/documents/nr-reporting-summary-flat.pdf

# Life sciences study design

All studies must disclose on these points even when the disclosure is negative.

| | |
|---|---|
| Sample size | Enrolment of 96 participants was estimated to give 90% power at 2.5% level of significance. A base rate of 10 severe hypoglycaemia episodes per year was assumed using data from the literature (refs 23 and 46 in the manuscript) and a final rate of 2 episodes per year after HARPdoc vs 3.8 in BGAT (the comparator). The sample size was then inflated to take account of within-group correlation and adjusted for therapist group, with the interclass correlation estimated as 0.02. Therapy groups were expected to have between 6 and 8 participants giving a design effect of 1 + 0.02 (8-1) = 1.14. We adjusted for having two end points (12 and 24 months) using Bonferroni correction (corrected alpha - 0.025%) |
| Data exclusions | For the intention to treat analysis all available data were used. Data were excluded from the per protocol analysis if the participants had not received the allocated intervention or started it more than 2 months after their originally scheduled course; if they failed the attendence described in the protocol as adherent (the first 3 days of each course, plus in the case of HARPdoc at least one individual session), were discovered after randomisation to have had an exclusion criterion (such as pregnancy) or undertook islet transplantation, or if their data had been collected outside the time window permitted by the protocol. |
| Replication | The study reports a non-CTIMP and data have not been replicated. In response to your comment (Please provide a rationale as to why data have not been replicated", we may not be understanding the question but we don't think it is expected that a randomised controlled trial such as this one, which compares two psycho-educational interventions for people with type 1 diabetes and treatment-resistant hypoglycaemia over a two year followup would normally be replicated prior to publication, or indeed necessarily precisely replicated in future work. |
| Randomization | Eligible people expressing an interest were invited to a screening visit and if found to be eligible and willing were enrolled and offered a face-to-face baseline data collection visit. Randomisation was conducted by an independent service at the King's Clinical Trials Unit (KCTU) once a minimum of 11 participants had been recruited, not more than one week prior to the intervention starting. Randomisation Groups of 11 - 16 participants were randomised at the level of the individual using block randomisation with fixed block size of 2, stratified by country of the site (UK or USA) and use of technology (yes or no to use of insulin pump and/or continuous glucose monitoring). |
| Blinding | Study subjects and educators delivering the interventions could not be blinded to the intervention, although both interventions were delivered by each centre in the same time frame. The trial statistician and the senior statistician remained blind to allocation until the final stages of the analysis, after all the data had been collected and cleaned. |

# Reporting for specific materials, systems and methods

We require information from authors about some types of materials, experimental systems and methods used in many studies. Here, indicate whether each material, system or method listed is relevant to your study. If you are not sure if a list item applies to your research, read the appropriate section before selecting a response.

## Materials & experimental systems

| n/a | Involved in the study | |
|---|---|---|
| ☒ | ☐ | Antibodies |
| ☒ | ☐ | Eukaryotic cell lines |
| ☒ | ☐ | Palaeontology and archaeology |
| ☒ | ☐ | Animals and other organisms |
| ☐ | ☒ | Human research participants |
| ☐ | ☒ | Clinical data |
| ☒ | ☐ | Dual use research of concern |

## Methods

| n/a | Involved in the study | |
|---|---|---|
| ☒ | ☐ | ChIP-seq |
| ☒ | ☐ | Flow cytometry |
| ☒ | ☐ | MRI-based neuroimaging |

## Human research participants

Policy information about studies involving human research participants

| | |
|---|---|
| Population characteristics | The population comprised 99 adults, aged (mean±SD) 54.3±13.3 years, 56% female, 96% white and with BMI 26.4 ±4.9 kg/m2, with diabetes duration 35.8±15.4 years, HbA1c 7.4±1.2% (57.3±13.1 mmol/mol). Mean rate of other significant medical conditions was 7.2±5.1. They had impaired awareness of hypoglycaemia (Gold score 5.5±1.2, Clarke score 5.4±1.1) with baseline rate of severe hypoglycaemia of (median [IQR] was 5[2-12] per patient/year. Mean rate of other medical conditions was All had received education in flexible insulin management, 84% in structured format. 85% had previously been offered some form of diabetes technology , with 55.6% using pump and/or CGM at baseline. |
| Recruitment | Potential participants were invited to express interest in the study by their diabetes health care professional. In some centres, an electronic data base was screened for possible eligibility (type 1 diabetes, report of severe hypoglycaemia and no listed exclusion) and eligible patients then invited to express interest. People expressing interest were then given information about the study, including the approved patient information sheets and an opportunity to discuss the study with a member of the research team. Those wishing to proceed were invited to sign a consent form and allocated a unique study number. They were then offered a screening visit that included documentation of experience of severe hypoglycaemia over the last 12 and 24 months and of hypoglycaemia awareness status. People who fulfilled all the eligibility criteria were then enrolled in the study. We deliberately targeted people with type 1 diabetes and problematic hypoglycaemia that had proven resistant to conventional therapies delivered in specialist care. People volunteering for this study had to be interested enough in addressing their problematic hypoglycaemia to volunteer but many were recommended to the programme by family members or health care professionals and had characteristics such as long duration of their type 1 diabetes in common with similar studies in the literature. Although we did not measure educational status, the requirement for fluency in English may have caused a bias. Our results may therefore not be applicable to people completely disinterested in their problematic hypoglycaemia and non-English speakers. |
| Ethics oversight | The protocol was approved by the London-Dulwich Research Ethics Committee (16/LO/1992) for the UK's Health Research Authority and the Committee on Human Studies of the Joslin Diabetes Center (2016-32) |

Note that full information on the approval of the study protocol must also be provided in the manuscript.

## Clinical data

Policy information about clinical studies

All manuscripts should comply with the ICMJE guidelines for publication of clinical research and a completed CONSORT checklist must be included with all submissions.

| | |
|---|---|
| Clinical trial registration | ClinicalTrials.gov identifier = NCT02940873 |
| Study protocol | The protocol can be accessed on the ClinicalTrials.gov website and a full description of the protocol has also been published (BMJOpen2019: 9: e0303356) |
| Data collection | The four study sites were: all secondary and tertiary diabetes service providers running structured education programmes in flexible insulin therapy and with access to diabetes technologies. They were: London, UK (Diabetes Services of King's College Hospital and the Guy's and St Thomas' NHS Foundation Trusts; the Diabetes Centre of the Northern General Hospital, Sheffield UK; the diabetes centre of the Royal Bournemouth Hospital in Dorset UK (part of the University Hospitals Dorset NHS Foundation Trust) and the Joslin Diabetes Center in Boston, MA, USA. The first participant was recruited in March 2017 and the first course started in Sept 2017. Recruitment was completed in March 2019. The final data collection was made in March 2021. |
| Outcomes | Primary outcome was the rate of severe hypoglycaemia events (number of events over the preceding year) measured using 12 and 24 month anonymised severe hypoglycaemia recall forms. Secondary endpoints included the number of events involving loss of consciousness or seizure, treated with IV glucose or IM glucagon, ambulance call outs;,Emergency Department attendances and admissions to hospital for at least one night, all in the last twelve months; number moderate hypoglycaemias in the last four weeks; glycated haemoglobin and the number of people whose glycated haemoglobin did not rise by more than 0.3%; and the following questionnaires, for which total and component factor scores were recorded: Attitudes to Awareness (measuring thoughts about hypoglycaemia); Hypoglycaemia Fear survey (HSF-II); the Hyperglycaemia Avoidance Score; the Problem Areas in Diabetes (PAID, measuring diabetes distress); the Hospital Anxiety and Depression scores, both as scores and also as people scoring 8 or more for either anxiety (HADS-A) or depression (HADS-D). The glycated haemoglobin was measured in blood taken either in a clinical phlebotomy service pr, after March 2020, collected from a finger prick by the participant at home (all samples measured centrally at the Viapath laboratory at King's College Hospital NHS Foundation Trust) and the questionnaires were completed in a paper booklet, with an online option after March 2020. |
